# Process Development for the Manufacture of the Antimalarial Amodiaquine Dihydrochloride Dihydrate

**DOI:** 10.1021/acs.oprd.3c00205

**Published:** 2023-12-18

**Authors:** Mukut Gohain, Modibo S. Malefo, Phaladi Kunyane, Chantal Scholtz, Sangeeta Baruah, Andile Zitha, Gerrit van der Klashorst, Hannes Malan

**Affiliations:** Department of Research and Development at Chemical Process Technologies (Pty) Ltd, 45 Battery Crescent, Waltloo, City of Tshwane, Gauteng 0184, South Africa

**Keywords:** antimalarial, amodiaquine dihydrochloride dihydrate, 4,7-dichloroquinoline, *meta*-chloroaniline, process

## Abstract

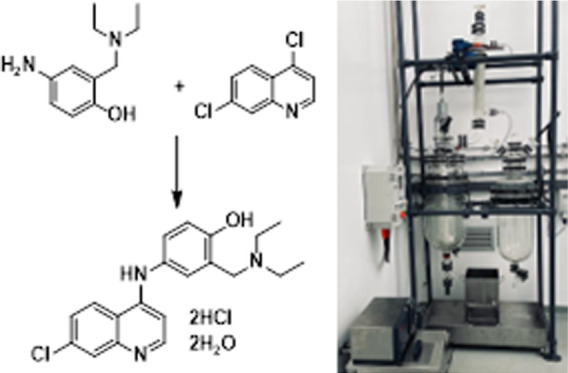

A robust process for the manufacture of the active pharmaceutical ingredient (API) amodiaquine dihydrochloride dihydrate (ADQ, **3**), an important antimalarial, is reported. The process consists of a three-step synthetic route that involves a Mannich reaction, substitution with 4,7-dichloroquinoline (4,7-DCQ, **5**), and rehydration. Additionally, a cost-competitive process for the production of 4,7-DCQ (**5**) is also reported wherein 4,7-DCQ (**5**) was prepared in four steps from *meta*-chloroaniline (**7**). 4-Acetamido-2-(diethylaminomethyl)phenol (**14**), 4,7-DCQ (**5**), and ADQ (**3**) were obtained in yields of 92, 89, and 90%, respectively. Costing and process mass intensities of 4,7-DCQ and ADQ are also reported.

## Introduction

Malaria is still one of the leading causes of death worldwide, with an estimated 247 million cases and 619,000 deaths reported in 2021.^1^ The main epidemic areas of malaria are distributed in Africa (96%), followed by Southeast Asia (SE Asia) (2%) and the Eastern Mediterranean Region (2%).^1^ The World Health Organization (WHO) hopes to eliminate malaria in at least 35 additional countries (based on data from 2021) by 2030.^2^

**Figure 1:**
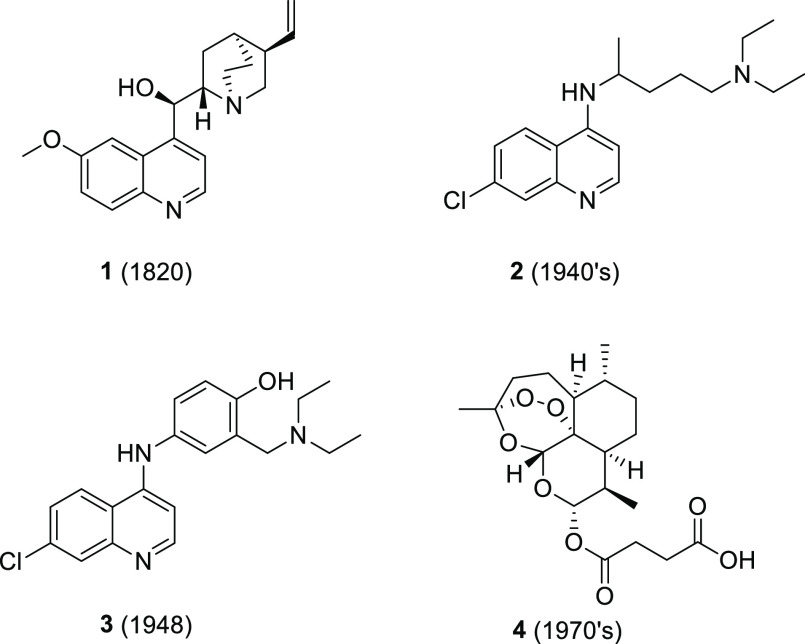
Structures of current antimalarial agents: quinine (**1**), chloroquine (**2**), amodiaquine (**3**), and artesunate (**4**).

Quinine (Figure 1, **1**) was the sole antimalarial drug used since its discovery in the 19th century, followed by chloroquine (**2**).^3^ However, due to emerging chloroquine resistance, more antimalarial drugs such as amodiaquine (ADQ, **3**) were discovered.^4,5^

ADQ (**3**), first discovered in 1948, is a 4-aminoquinoline antimalarial drug used (in base or acid form) as an alternative against chloroquine-resistant strains.^6−8^ Due to the severe side effects from the sole use of ADQ (**3**), the WHO has recommended the implementation of artemisinin-based combination therapy (ACT), which is the pairing of ADQ (**3**) with artemisinin derivatives such as artesunate (**4**) as a first-line of treatment for uncomplicated malaria.^9,10^ A 2006 study on the use of ACT in a village in Uganda concluded that the use of ACT offered an important step forward for the treatment of malaria in Africa and that more extensive research into the development of a cost-effective ACT and coformulations is a necessity.^11^

Despite the availability of antimalarial drugs such as amodiaquine (**3**), most of sub-Saharan Africa still lacks adequate access to good quality, affordable antimalarial drugs due to most active pharmaceutical ingredients (APIs) being imported from other countries. While published processes are available for ADQ and 4,7-DCQ,^9,10,12−20^ each of these methods has limitations such as low yields, the formation of impurities, and the use of expensive or hazardous solvents, and more unit operations are required for the production of amodiaquine, which drives up the energy requirements.^9,21^ Additionally, no methods are reported for the removal of impurities or for the exact determination of the crystal water molecules in ADQ.^12^ These limitations make it difficult to produce ADQ (**3**) at a competitive price.

Process development entails the development, optimization, and scale-up of a chemical synthetic route that can be transferred into a cost-effective, safe, and reproducible manufacturing process. The development comprises three stages: bench-scale, kilo-scale, and pilot-plant scale, with process validation at each stage.^22,23^ During the initial stages, the most robust synthetic route is investigated, optimized, and validated, followed by scale-up of the chosen route to kilogram scale in the kilo lab.^24^ Key factors that are considered during each stage include the temperature of the reaction, reaction time, number of steps, product loss minimization, workup and product isolation procedures, waste management and environmental impact, reproducibility, and costs involved. When the above-mentioned are satisfied, the process is transferred to the pilot plant where aspects such as scalability, safety, and quality are further evaluated. In each stage of development, the total process cost is measured, which ultimately contributes to the total API product cost (material cost + conversion cost).^22,23^ In addition, the process mass intensity is also calculated to determine the efficiency of the developed processes.^25^

## Results and Discussion

4,7-Dichloroquinoline (**5**) is an important component of several antimalarial drugs^15,26^ and is therefore a major driver of cost in the production of amodiaquine (**3**) as it accounts for over 40% of the raw material costs. Thus, a robust, cost-competitive process for the production of amodiaquine (**3**) would require preferred pricing from commercial suppliers. This, however, would be a temporary solution as there would be no internal control of costs; hence, an equally cost-competitive process for the manufacture of 4,7-dichloroquinoline (**5**) is needed to be developed in-house for continuous raw material supply without interruption during ADQ (**3**) production.

### Preparation and Process Development of 4,7-Dichloroquinoline (**5**)

**Scheme 1:**
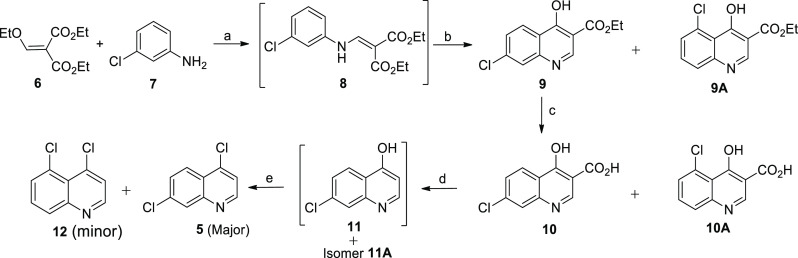
Synthesis of 4,7-Dichloroquinoline (**5**) Reagents and conditions: (a) 100 °C, 2 h; (b) DPE, 250 °C, 2 h; (c) 10% aq NaOH, 2 h, 10% aq H_2_SO_4_; (d) DPE, 250 °C, 2 h; and (e) 135 °C, POCl_3_, 2 h.

The process for the commercial production of 4,7-dichloroquinoline (**5**) was developed according to the methodology of Price and Roberts (Scheme 1),^15^ which is centered on diethoxymethylene malonate (**6**) and *meta*-chloroaniline (**7**), with necessary modifications.

**Figure 2:**
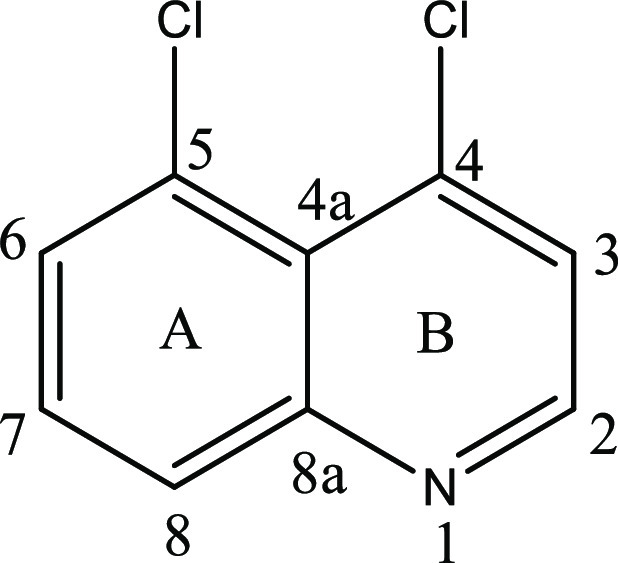
4,5-Dichloroquinoline (**12**).

The synthesis of 4,7-dichloroquinoline (**5**) commenced by the conjugate addition of diethoxymethylene malonate (**6**) and *meta*-chloroaniline (**7**) to afford the acrylate intermediate **8**, which, upon thermal cyclization in diphenyl ether (DPE), afforded the quinoline ester **9** in good yields (90–96%). Hydrolysis of the ester (**9**) in aqueous sodium hydroxide to the quinoline acid (**10**) was achieved in essentially quantitative yields, while thermal decarboxylation and subsequent chlorination with POCl_3_ gave the target product **5** in 81–90% yields. The GC–MS chromatogram (Figure 2) of the crude product showed an extra peak at a retention time of 13.13 min with a similar mass to that of 4,7-dichloroquinoline (**5**).^15^ After isolation and characterization using 1D and 2D NMR, the identity of the impurity was confirmed to be the 4,5-dichloroquinoline isomer (**12**, Figure 2).^20^ The ^1^H NMR spectrum of isomer **12** displayed two doublets integrating for one proton each at δH 8.72 (d, *J* = 4.68 Hz, 1H, H-2) and at δH 7.52 (d, *J* = 4.68 Hz, 1H, H-3) for the protons on the B-ring. An ABX spin system was observed at δH 8.05 (dd, *J*_1_ = 8.32 Hz, *J*_2_ = 1.44 Hz, H-8); 7.67 (dd, *J*_1_ = 7.56 Hz, *J*_2_ = 1.42 Hz, 1H, H-6); and 7.60 (dd, *J*_1_ = 7.96 Hz, *J*_2_ = 7.96 Hz, 1H, H-7) for the A-ring aromatic protons. Additional distinguishing NMR correlations were observed in the COSY NMR spectrum, with the proton–proton correlation between the three protons H-6, H-7, and H-8 confirming that they were adjacent to each other. The ortho–meta (7.56, 1.42 Hz) coupling constants for H-6, ortho–ortho (7.96, 7.96 Hz) coupling constants for H-7, and ortho–meta (8.32, 1.44 Hz) coupling constants for H-8 provide further confirmation of the arrangement of these protons.

Attempted recrystallization in hexane, as reported by Price and Roberts,^15^ afforded the pure product in moderate yields of 65% (Table 1, entry 1). Several solvents and conditions were tried in order to improve the yields, with recrystallization in heptane, resulting in a slight improvement in yields without compromising the purity (entry 2). In contrast to the alkanes, OH-containing solvents such as ethanol and methanol resulted in a drastic decrease in yield (entries 3 and 5). In an attempt to minimize the solubility of the product, 5% water was added to the recrystallization; this, however, showed an inverse relationship between purity and yields as the purity significantly reduced from 99.5 to 86 and 90% for MeOH/H_2_O and EtOH/H_2_O, respectively.

**Table 1:** Recrystallization of Crude 4,7-Dichloroquinoline (**5**)

entry	solvent	yield (%)	purity by GC (%)
1	hexane	65	99.5
2	heptane	67	99.5
3	EtOH	56	99.5
4	EtOH/H_2_O	75	90
5	MeOH	60	99.5
6	MeOH/H_2_O	73	86

Even though successful in its objective of removing the 4,5-dichloroquinoline isomer (**12**), the loss in yield and a need for an extra step outweighed the advantages. Another option to remove the isomer would be to take advantage of solubility due to the difference in the acidity of the two chloroquinoline acid isomers during hydrolysis.

Acetic acid has been reported^27^ as an excellent solvent for the selective precipitation for the isolation of the two quinoline acid isomers (**10** and **10A**); however, the harsh conditions (reflux) significantly reduce the selectivity, resulting in lower yields of the quinoline acid (**10**). Additionally, it is expensive and would require extra care in the plant due to its strong odor and harmful effects; thus, using acetic acid as the solvent for the elimination of the 4,5-isomer (**12**) would not be ideal on a commercial scale.

**Figure 3:**
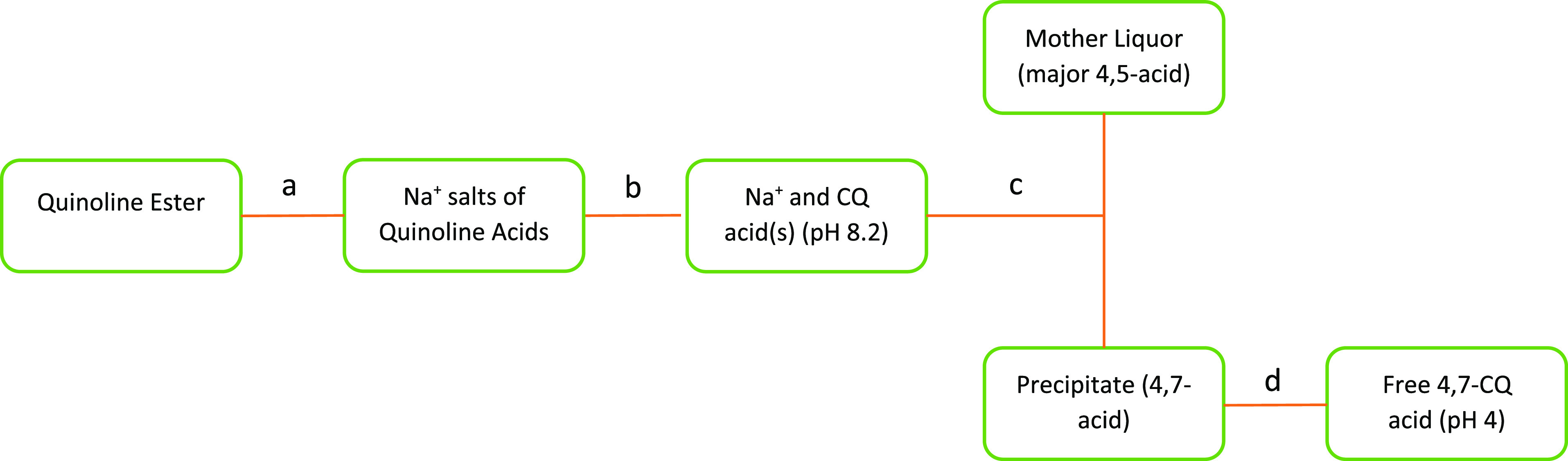
Fractional precipitation flow diagram consisting of (a) hydrolysis with NaOH; (b) neutralization; (c) filtration; and (d) slurry-wash at pH 4. (CQ—chloroquinoline acid.)

The reported pH for the precipitation of the quinoline acid (**10**) is Congo red (pH 5),^15^ which upon decarboxylation and chlorination affords 4,7-dichloroquinoline (**5**) with 3–4% of the 4,5-dichloroquinoline isomer (**12**). In this study, we envisaged that, owing to the difference in acidity, the two isomers, or at least the majority thereof, would precipitate at different pH values and thus be isolable (Figure 3). Upon hydrolysis of **9** with NaOH, the resulting mixture is basified to pH 8.2–8.4, instead of pH 4 as reported in the literature. The precipitate is then isolated by filtration and slurry-washed at pH 4 to remove any remaining sodium salt. The resulting quinoline acid is then subjected to decarboxylation and chlorination with POCl_3_ to afford the target product 4,7-dichloroquinoline (**5**) with high purity.

We began our investigation by precipitating the quinoline acid (**10**) at pH 6.5, which afforded the acid in almost quantitative yields (98%, Table 2, entry 1). However, GC assay results indicated that the final product was contaminated with the isomer (Supporting Information, Figure S1).

**Table 2:** Effect of Changing pH on the Purity of 4,7-DCQ (**5**)

entry	temperature (°C)	pH	isolated yield (%)	outcome observed in GC^a^ (Area %)
1	45	6.5	99	4,7-DCQ (95.64%) and 4,5-DCQ (4.36%)
2	45	7.0	97	4,7-DCQ (97.23%) and 4,5-DCQ (2.77%)
3	45	7.5	95	4,7-DCQ (97.65%) and 4,5-DCQ (2.35%)
4	45	8.0	92	4,7-DCQ (97.96%) and 4,5-DCQ (2.04%)
5	45	8.10	91	4,7-DCQ (98.90%) and 4,5-DCQ (1.10%)
6	45	8.20	90	4,7-DCQ (100%) (Figure S2)
7	45	8.5	84	4,7-DCQ

a Area % calculated by GC and identified by GC–MS.

Nonetheless, as the pH was increased from 7.0 to 7.5, the isomer in the isolated product decreased (entries 2 and 3). Similarly, at pH 8.0–8.10 (entries 4 and 5), only trace amounts of the isomer were observed. At pH 8.2 and 8.5 (entries 6 and 7), there was virtually no isomer observed in the spectrum (Figure S2), affording the target product in 99.3% purity by GC assay. Although the yields decreased from 99% (entry 1) to 90% (entry 6), the fractional precipitation technique proved superior to solvent recrystallization, where yield losses of up to 25% were observed. Additionally, this is a single-operation process and does not require extra solvent. Moreover, the reactions were first attempted at room temperature at which a thick heterogeneous slurry formed and rendered the mixture difficult to stir. However, as the temperature was increased to 45 °C, the mixture was homogeneous and easy to stir and filter, which ultimately afforded the desired purity.

The optimized process described above was demonstrated to be repeatable in more than 20 experiments varying in scale from 15 to 500 g. A 4,7-DCQ (**5**) yield of 90% and a purity of more than 97% were achieved throughout. This process resulted in an overall yield of 75% of the correct quality 4,7-DCQ (**5**). Based on this, the material cost per kilogram for 4,7-DCQ (**5**) was calculated as $24.15 *versus* a market price of $42.00/kg. The material margin of the process at current market prices is slightly better at 42% *versus* the target material margin of 37% specified in the scope of this project. The process mass intensity (PMI) of the 4,7-DCQ process was also calculated, which was found to be 9.65 kg of raw materials required to produce per kg of 4,7-DCQ after recycling of water and solvents (Supporting Information, entry no. 2). The low PMI indicated that the developed process was highly efficient for the commercial manufacture of 4,7-DCQ. That said, the successful development of a cost-effective, competitive process for the production of 4,7-dichloroquinoline (**5**) would mitigate the reliance on imports and provide a steady supply of this critical intermediate.

### Preparation of Amodiaquine Dihydrochloride Dihydrate (**3**)

**Scheme 2:**
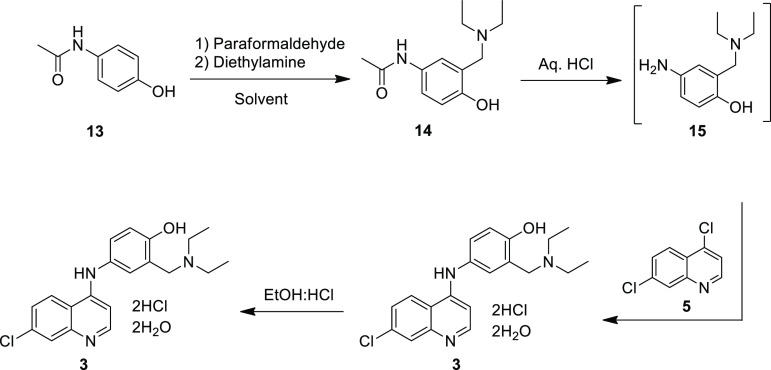
Preparation of Amodiaquine Dihydrochloride Dihydrate (**3**) from 4-Acetamidophenol (**13**)

The synthesis of amodiaquine dihydrochloride dihydrate (**3**) was performed as shown in Scheme 2 following the reported procedure by Burckhalter *et al.*(12) with slight modifications. Amodiaquine dihydrochloride dihydrate (**3**) was prepared *via* a 4-step synthetic scheme involving a Mannich reaction, followed by hydrolysis of the amide group and a subsequent substitution with 4,7-dichloroquinoline (**5**). The key intermediate **14** was prepared by subjecting 4-acetamidophenol (**13**) to a Mannich reaction with diethylamine (DEA) and paraformaldehyde in a solvent. Several reaction conditions were attempted before achieving a robust method to obtain the Mannich base (**14**) in desirable yields.

The first attempt followed the reported procedure where paraformaldehyde was reacted with DEA in methanol for 2 h at 40 °C to allow for the formation of the iminium ion, followed by the addition of 4-acetamidophenol (**13**) and stirring of the reaction at 64 °C for 3 h (Table 3). However, TLC analysis showed incomplete conversion of **13** within 3 h; thus, the reaction was continued for 24 h while monitoring progress at intervals of 2 h to afford the Mannich base (**14**) in a moderate yield of 60%. With the intention of reducing the reaction time, the reaction was repeated in methanol and 32% HCl; however, within 7 h, TLC analysis showed the formation of unidentifiable impurities. The following attempts ran the Mannich reaction in acetic acid at varying temperatures from 50 to 80 °C for 5–24 h. The reaction proceeded well at lower temperatures but slowly. As the reaction temperature was increased, more impurities, which were attributed to the double-Mannich reaction,^5^ were formed instead.

**Table 3:** Varying Reaction Conditions for the Preparation of the Mannich Base (**14**)

entry	solvent	temperature (°C)	time (h)	yield (%)
1	methanol	65	3	82
2	methanol	60	24	60
3	ethanol	78	15	poor conversion
4	isopropanol	85	24	87
5	methanol + HCl	60	7	
6	isopropanol + *p*-TSA	85	24	61
7	AcOH	50/80	5–24	
8	toluene	85	15	95

The reaction was then attempted in ethanol at 78 °C, which, within 15 h, had proceeded poorly. Isopropanol was the next solvent attempted at 85 °C, which showed improved yields of 87% after 24 h with minimal impurity formation. To catalyze the reaction, with the aim of reducing the reaction time, the reaction was repeated in isopropanol in the presence of *p*-toluenesulfonic acid (*p*-TSA) as a catalyst (entry 6). However, TLC analysis showed the formation of more impurities than those observed in the earlier attempt (entry 4), and the Mannich base (**14**) was obtained in reduced yields of 61% (*versus* 87%). It was clear at this point that the use of any acid promoted the formation of more impurities. The next attempt saw the reaction performed in toluene at 85 °C, which afforded **14** in an excellent yield of 95% within 15 h. Moreover, to the best of our knowledge, a C–C bond formation Mannich reaction in toluene has not been reported in the literature previously.^28^ Due to toluene’s relative affordability and its ability to be recycled and reused, this contributes to cost-cutting and ultimately renders our process competitively cheaper.

Having successfully developed the process for the preparation of the Mannich base (**14**), the next step was to synthesize the final product **3**. The synthesis of ADQ (**3**) was carried out in two steps from the intermediate **14**, following the reported procedure, which involved hydrolysis of the Mannich base followed by substitution with 4,7-DCQ (**5**) *in situ*.^12^ As with the preparation of the Mannich base, several reaction conditions were examined to find a robust process for the preparation of ADQ (**3**).

For the first attempt, the Mannich base (**14**) was refluxed in 20% HCl for 4 h at 80 °C followed by distillation of the excess HCl and then substitution with 4,7-DCQ (**5**) in ethanol for 24 h to give ADQ (**3**) in 43% yield. In addition to the low yield obtained, this process required extra energy to distill out water from the reaction; thus, it would not be practical during the scale-up of the process. The next attempt involved refluxing the Mannich base (**14**) in a mixture of HCl/H_2_O/solvent for 3–5 h, where the solvent was either ethanol or isopropanol, followed by substitution with 4,7-DCQ (**5**). Table 4 entry 2 shows that the reaction in ethanol produced a low yield of 10%, whereas isopropanol (entry 3) resulted in an improved yield of 58%. When the same reaction conditions were attempted in the absence of an organic solvent (entry 4), a yield of 53% was obtained. The next attempt involved subjecting the Mannich base (**14**) to hydrolysis in commercial-grade HCl (32%) at 85 °C for 4 h to produce the amine (**15**), which, after pH adjustment to 4, was reacted with 4,7-DCQ (**5**) *in situ* to afford the desired amodiaquine dihydrochloride dihydrate (**3**). Crude **3** was recrystallized from ethanol and rehydrated by refluxing in water followed by precipitation at cool conditions to obtain amodiaquine dihydrochloride dihydrate (**3**) in an excellent yield of 90% with USP quality. The HPLC chromatogram shows only a single peak at a retention time between 5 and 6 min, proving the absence of starting material (Figures S3–S5).

**Table 4:** Reaction Conditions for the Synthesis of Amodiaquine Dihydrochloride Dihydrate (**3**)

entry	hydrolysis conditions	substitution conditions	yield (%)
1	20% HCl, 80 °C, 4 h	EtOH, 24 h, 78°C	43
2	32% HCl (9 mL), H_2_O (9 mL), EtOH (7.4 mL), 3 h	3 h, 78°C	10
3	32% HCl (9 mL), H_2_O (9 mL), IPA (7.4 mL), 80°C, 2.5 h	2 h, 80°C	58
4	32% HCl (5 mL), H_2_O (5 mL), 80°C, 5 h	15 h, 80°C	53
5	32% HCl, 80–85°C, 4 h, H_2_O	3 h, 80–85°C	90

Once a robust synthetic route suitable for the manufacturing process was developed and optimized, the next step was to prove its scalability and reproducibility. This was done by following the developed route on a 100–400 g and 5 kg scale at least three times (100–300 g) as shown in Table 5 and analyzing the intermediates and products by GC–MS, IR, NMR, MP, and HPLC. There were no significant changes required to the route on a 500 g scale; however, as the scale was increased to 5 kg, temperature and time became the major optimization points. The larger the reactor, the longer it took to heat up the reaction as required; therefore, heating the reaction and subsequently cooling to adjust the pH after hydrolysis of the Mannich base (**14**) followed by substitution with 4,7-DCQ (**5**) at 90 °C was not feasible. It then became necessary to adjust the pH under hot conditions at 50 °C, which did not cause any problems or impurity formation despite our concerns.

**Table 5:** Reproducibility of the Developed Process at Different Reaction Scales

product	scale (g)	yield for repetition 1 (%)	yield for repetition 2 (%)	yield for repetition 3 (%)
Mannich base (**14**)	150	92	93	92
Mannich base (**14**)	390	94	95	94
ADQ (**3**)	200	90	91	90
ADQ (**3**)	300	90	89	91

The overall yield for the optimized process is 86% when starting from 4-acetamidophenol (**13**). Based on quotes obtained from bulk chemical suppliers during the process development, the material cost for ADQ (**3**) has been calculated as $16.57/kg (Supporting Information, entry no. 2), resulting in a material margin of 59%. The key raw material cost drivers are diethyl ethoxymethylene malonate (**6**) and 4-acetamidophenol (**13**). The raw material margin of 59% is slightly better than the target margin of 57% defined in the scope of this project. The lower raw material margin allows for additional costs such as conversion costs, labor, and depreciation. The PMI for the ADQ (**3**) process was found to be 3.34, which indicated that the developed process was very efficient for the production of ADQ (**3**) (Supporting Information, entry no. 2). It can thus be concluded that the known process for the manufacture of amodiaquine (**3**) and its key intermediate 4,7-dichloroquine (**5**) was optimized, resulting in a lower raw material cost with a potential reduction of the selling price. In conclusion, 4,7-dichloroquinoline (**5**), 4-acetamido-2-(diethylaminomethyl)phenol (**14**), and amodiaquine dihydrochloride dihydrate (**3**) were synthesized on a kilogram scale, as part of the current project, resulting in the successful development of a robust, efficient, economically competitive, scalable, and reproducible process that can be transferred to a commercial process for the manufacture of the antimalarial API amodiaquine dihydrochloride dihydrate (**3**).

## Experimental Section

### General Experimental Procedure

All of the raw materials and solvents purchased were used without further purification. Thin layer chromatography (TLC) was performed on Macherey-Nagel 0.2 mm silica gel 60 F254 packed aluminum plates observed under UV light at 254 nm. The synthesized compounds were analyzed by FT-IR spectroscopy, NMR spectroscopy with the residual solvent peak as an internal reference (DMSO-*d*_6_ = 2.50 and 39.5 ppm and CDCl_3_ = 7.26 and 77.16 ppm for ^1^H and ^13^C NMR spectra, respectively), and gas chromatography mass spectroscopy (GC–MS). The purity of the final product **3** was determined by using high-performance liquid chromatography (HPLC) on a Hitachi system equipped with a LUNA C18 column and a diode array detector set at 224 nm.

Thermal analyses of the final ADQ products (**3**) were conducted using thermogravimetric analysis (TGA), TGA-TA 5500, and differential scanning calorimetry (DSC), DSC-TA 2500, under a nitrogen atmosphere. The TGA and DSC thermograms were analyzed by TRIOS 5.3.0.48151 version and Origin2018. Isothermal experiments were performed with a TRIOS 5.3.0.48151 version calorimeter with a nitrogen flow rate of 50 mL/min.

#### 3-Carbethoxy-7-chloro-4-hydroxyquinoline (**9**)

520 g portion (2.40 mol, 1.1 equiv) of diethoxymethylene malonate was added to *meta*-chloroaniline (300 g, 2.35 mol, 1.0 equiv), and the reaction mixture was heated under stirring at 97–100 °C for 2 h. Under a nitrogen atmosphere, the warm acrylate was added dropwise into a hot solution of diphenyl ether (225 °C). The reaction mixture was stirred at 225 °C for 2 h followed by cooling to 50 °C. 1000 mL of toluene was added to the semisolid mass, and the mixture was stirred well for 15 min. The product was filtered *in vacuo*, washed with toluene (2 × 500 mL), and dried in the oven (40 °C) to afford **9** as a brown fluffy solid (540 g, 2.41 mol, 91%). mp = 294–296 °C (lit. = 295–297 °C).^29^

#### 7-Chloro-4-hydroxyquinoline-3-carboxylic Acid (**10**)

To a stirred solution of aq NaOH (25%, 2000 mL), the quinoline ester **9** (540 g, 2.41 mol, 1.0 equiv) was added. The reaction mixture was heated at 95–97 °C for 2 h, during which all the ester dissolved to form a brown solution. The reaction mixture was cooled to room temperature and neutralized to pH 8.2 by the slow addition of a 10% aq H_2_SO_4_ solution. The reaction mixture was heated at 45 °C for 1 h (maintaining the pH 8.2). The precipitate was collected by filtration while warm and washed with water (2 × 200 mL). The filter cake was suspended in 2500 mL of water and stirred vigorously, and the pH was adjusted to 4 by the slow addition of a 10% aq H_2_SO_4_ solution. The quinoline acid was filtered *in vacuo*, bed-washed with 2000 mL of H_2_O, and dried in a vacuum oven (100 °C) overnight to afford **10** as a white powder (430 g, 1.20 mol, 89%). mp = 272–273 °C (lit. = 273–274 °C).^29^

#### 4,7-Dichloroquinoline (**5**)

To a stirred solution of diphenyl ether (2381 mL) under a nitrogen atmosphere, quinoline acid (**10**, 500 g, 2.236 mol, 1.0 equiv) was added. The reaction mixture was refluxed at 225–227 °C for 4 h, during which all the solids dissolved to form a light brown solution. The reaction mixture was cooled gradually to 30 °C, and then 221.4 mL (1.1 equiv) of POCl_3_ was added dropwise over 10 min. The reaction mixture was refluxed at 133–135 °C for 2 h and then cooled to 30 °C, and 50 mL of toluene was added. The organic layer was extracted three times at 80 °C with 100 mL of 10% aq HCl. The combined aqueous layers were washed with 100 mL of toluene and then chilled to 15 °C. The pH was adjusted to 0.5 with 25% aq NaOH, and the resulting brown precipitate was filtered off. The mother liquor was basified to pH 12.6 by the slow addition of a 25% aq NaOH solution, and the product was collected by filtration under vacuum and slurry-washed with 3571 mL of water. The product was filtered *in vacuo* and dried in an oven at 40 °C for 48 h to afford **5** as a cream white solid (397.6 g, 2.008 mol, 90%). mp = 84–85 °C (lit. = 84–86 °C).^28^^1^H NMR (400 MHz, CDCl_3_): δ 8.77 (d, *J* = 4.72 Hz, 1H, H-2); 8.15 (d, *J* = 8.96 Hz, 1H, H-5); 8.10 (d, *J* = 2.04 Hz, 1H, H-8); 7.57 (dd, *J*_1_ = 8.96 Hz, *J*_2_ = 2.08 Hz, 1H, H-6); 7.47 (d, *J* = 4.72 Hz, 1H, H-3). ^13^C NMR (100 MHz, CDCl_3_): δ 151.11; 149.55; 142.80; 136.63; 128.87; 128.77; 125.71; 125.12; 121.53. HRMS *m*/*z*: [M + H]^+^ 197.040 (calculated for C_9_H_5_Cl_2_N 198.057).

#### 4,5-Dichloroquinoline (**12**)

Isolated from the crude product by column chromatography, eluting with EtOAc/hexanes (1:9 to 3:7 v/v) affords isomer **12** as a white crystalline solid. mp = 115.7–116.4 °C (lit. = 116–117 °C).^20^^1^H NMR (400 MHz, CDCl_3_): δ 8.72 (d, *J* = 4.68 Hz, 1H, H-2); 8.05 (dd, *J*_1_ = 8.32 Hz, *J*_2_ = 1.44 Hz, 1H, H-8); 7.67 (dd, *J*_1_ = 7.56 Hz, *J*_2_ = 1.42 Hz, 1H, H-6); 7.60 (dd, *J*_1_ = 7.96 Hz, *J*_2_ = 7.96 Hz, 1H, H-7); 7.52 (d, *J* = 4.68 Hz, 1H, H-3). ^13^C NMR (100 MHz, CDCl_3_): δ 151.30; 149.97; 141.70; 131.09; 130.26; 130.09; 129.61; 125.09; 124.03. HRMS *m*/*z*: [M + H]^+^ 199.039 (calculated for C_9_H_5_Cl_2_N 198.057).

#### 4-Acetamido-2-(diethylaminomethyl)phenol (**14**)

To a solution of paraformaldehyde (119.22 g, 3.97 mol, 1.2 equiv) in toluene (2000 mL) was added diethylamine (302 g, 4.13 mol, 1.25 equiv) dropwise. The mixture was stirred for 2 h at 40 °C before adding 4-acetamidophenol (500 g, 0.331 mol, 1.0 equiv) to the mixture followed by stirring for 15 h at 80–85 °C. The mixture was gradually cooled to room temperature and subsequently stirred for 2 h at 5–10 °C. The product was filtered *in vacuo*, washed with toluene (2 × 500 mL) and water (500 mL), and dried in the oven at 40 °C to afford **14** (740 g, 3.14 mol, 95%) as a white powder: mp = 133.3–135.5 °C (lit. = 135 °C).^12^^1^H NMR (500 MHz, DMSO-*d*_6_): δ 9.63 (s, 1H, NH); 7.28 (s, 1H, Ar-H); 7.25 (d, *J* = 8.60 Hz, 1H, Ar-H); 6.60 (d, 1H, *J* = 8.60 Hz, Ar-H); 3.65 (s, 2H, CH_2_NEt_2_); 3.56 (s, 1H, OH); 2.53 (q, 4H, *J* = 7.10 Hz, NCH_2_CH_3_); 1.97 (s, 3H, AcCH_3_); 1.01 (t, 6H, *J* = 7.15 Hz, N(CH_2_CH_3_)_2_). ^13^C NMR (125 MHz, DMSO-*d*_6_): δ 167.45; 153.19; 130.82; 122.61; 120.00; 119.34; 115.05; 55.23; 45.80; 23.73; 11.11. HRMS *m*/*z*: [M + H]^+^ 237.186 (calculated for C_13_H_21_N_2_O_2_ 236.16).

#### Amodiaquine Dihydrochloride Dihydrate (**3**)

4-Acetamido-2-(diethylaminomethyl)phenol (**14**, 400 g, 1.696 mol, 1.0 equiv) was added to a flask charged with 32% HCl (880 mL, 7.728 mol). The mixture was stirred for 15 min at room temperature followed by reflux at 85 °C for 4 h. H_2_O (1600 mL) was added to the flask, the heating was turned off, and the temperature was allowed to cool to 50 °C. The pH of the mixture was adjusted to 4 using a 25% aq NaOH solution. 4,7-DCQ (**5**, 336 g, 1.696 mol, 1.0 equiv) was added to the mixture. The mixture was then refluxed at 85 °C for 3 h, followed by stirring the mixture at 5 °C for 2 h. The yellow product was collected by vacuum filtration and washed with water (2 × 400 mL). The crude amodiaquine was kept under vacuum for 30 min after which it was refluxed in a 2400 mL solution of EtOH/HCl (5:1 equiv) for 2 h at 80 °C. The yellow product was then allowed to precipitate at 5–10 °C for 2 h at which time it was filtered *in vacuo*, washed with an 800 mL cold solution of EtOH/HCl (5:1 equiv), and air-dried overnight. The product was then refluxed in water (2.5 mL/g) at 95 °C for 2 h, followed by precipitation overnight at room temperature under stirring. The reaction mixture was cooled to 0–5 °C for 2 h. The product was filtered *in vacuo*, washed with cold water (2 × 400 mL), and air-dried overnight before drying in the oven at 40 °C to obtain **3** (708 g, 1.520 mol, 90%) as a yellow solid. HPLC (C18) *P*_HPLC_ 100%, *t*_R_ 7.3 min. mp = 159–166 °C (lit. = 160 °C).^12^ Water content = 8% (USP standard = 7.0–9.0%). ^1^H NMR (400 MHz, DMSO-*d*_6_): δ 14.88 (br s, 1H, OH); 11.22 (s, 1H, NH); 10.92 (br s, 1H, NH); 10.34 (br s, 1H, NH); 8.94 (d, 1H, *J* = 9.20 Hz, Ar-H); 8.47 (d, 1H, *J* = 7.08 Hz, Ar-H); 8.19 (d, 1H, *J* = 2.08 Hz, Ar-H); 7.83 (dd, 1H, *J*_1_ = 2.10 Hz, *J*_2_ = 9.10 Hz, Ar-H); 7.69 (d, 1H, *J* = 2.56 Hz, Ar-H); 7.37 (dd, 1H, *J*_1_ = 2.60 Hz, *J*_2_ = 8.68 Hz, Ar-H); 7.22 (d, 1H, *J* = 8.68 Hz, Ar-H); 6.84 (d, 1H, *J* = 7.04 Hz, Ar-H); 4.24 (s, 2H, CH_2_); 3.11 (s, 4H, 2× NCH_2_CH_3_); 1.29 (t, 6H, *J* = 7.20 Hz, 2× NCH_2_CH_3_). ^13^C NMR (100 MHz, DMSO-*d*_6_): δ 156.11; 154.95; 143.09; 138.97; 138.25; 130.13; 128.45; 127.95; 127.20; 126.18; 117.65; 116.87; 115.67; 100.45; 49.14; 46.19; 8.45. HRMS *m*/*z*: [M + H]^+^ 356.189 (calculated for C_20_H_22_ClN_3_O, 356.157).

### HPLC Method

The purity of the final product **3** was determined by HPLC using the LUNA C18 column on a Hitachi system equipped with a diode array detector set at 224 nm. The HPLC method followed that of the USP method for amodiaquine hydrochloride. Compounds were dissolved in water (15 mg/100 mL) and injected through a loop. Retention time (*t*_R_) was obtained at a flow rate of 1.2 mL/min using an isocratic run of 78% eluent A (potassium phosphate buffer) and 22% eluent B (MeOH) for a period of 0 to 15 min. The purity of the sample was determined based on the pharmacopoeia standard by preparing two standard solutions (15 mg in 100 mL of water), one with six injections and the other with two injections, to determine standard recovery with acceptable criteria of 97–103. After different drying conditions were evaluated, the oven-dried product (15 mg) was dissolved in 100 mL of water and injected (10 μL) into the specified column with a runtime of 15 min.

## Supporting Information Available

Copies of GC chromatograms, UV spectrum, HPLC chromatogram and report, FT-IR spectrum, thermogravimetric analysis data, NMR spectra, costing calculations, and PMI calculations (PDF)

## References

[ref1] WHO. World Malaria Report 2022; World Health Organization: Geneva, 2022.

[ref2] WHO. Global Technical Strategy for Malaria 2016–2030; World Health Organization: Geneva, 2015.

[ref3] Achan J.; Talisuna A. O.; Erhart A.; Yeka A.; Tibenderana J. K.; Baliraine F. N.; Rosenthal P. J.; D’Alessandro U. Quinine, an old anti-malarial drug in a modern world: role in the treatment of malaria. Malar. J. 2011, 10 (1), 144 10.1186/1475-2875-10-144. 21609473 PMC3121651

[ref4] Rajapakse C. S. K.; Lisai M.; Deregnaucourt C.; Sinou V.; Latour C.; Roy D.; Schrével J.; Sánchez-Delgado R. A. Synthesis of New 4-Aminoquinolines and Evaluation of Their In Vitro Activity against Chloroquine-Sensitive and Chloroquine-Resistant Plasmodium falciparum. PLoS One 2015, 10 (10), e0140878 10.1371/journal.pone.0140878. 26473363 PMC4608832

[ref5] Delarue-Cochin S.; Paunescu E.; Maes L.; Mouray E.; Sergheraert C.; Grellier P.; Melnyk P. Synthesis and antimalarial activity of new analogues of amodiaquine. Eur. J. Med. Chem. 2008, 43 (2), 252–260. 10.1016/j.ejmech.2007.03.008. 17485145

[ref6] Brasseur P.; Guiguemde R.; Diallo S.; Guiyedi V.; Kombila M.; Ringwald P.; Olliaro P. Amodiaquine remains effective for treating uncomplicated malaria in west and Central Africa. Trans. R. Soc. Trop. Med. Hyg. 1999, 93 (6), 645–650. 10.1016/S0035-9203(99)90083-4. 10717757

[ref7] Winstanley P. A.; Simooya O.; Kofi-Ekue J. M.; Walker O.; Salako L. A.; Edwards G.; Orme M. L.; Breckenridge A. M. The disposition of amodiaquine in Zambians and Nigerians with malaria. Br. J. Clin. Pharmacol. 1990, 29 (6), 695–701. 10.1111/j.1365-2125.1990.tb03690.x. 2378788 PMC1380171

[ref8] Wu R.; Williams R. F.; Silks L. P.; Schmidt J. G. Synthesis of stable isotope-labeled chloroquine and amodiaquine and their metabolites. J. Label. Compd. Radiopharm. 2019, 62 (5), 230–248. 10.1002/jlcr.3721. 30882940

[ref9] Fortunak J. M.; Kulkarni A. A.; King C. Green chemistry synthesis of the malaria drug amodiaquine and analogs thereof. WO 2013138200 A1, 2013.

[ref10] Mutabingwa T. K.; Anthony D.; Heller A.; Hallett R.; Ahmed J.; Drakeley C.; Greenwood B. M.; Whitty C. J. M. Amodiaquine alone, amodiaquine+sulfadoxine-pyrimethamine, amodiaquine+artesunate, and artemether-lumefantrine for outpatient treatment of malaria in Tanzanian children: a four-arm randomised effectiveness trial. Lancet 2005, 365 (9469), 1474–1480. 10.1016/S0140-6736(05)66417-3. 15850631

[ref11] Bukirwa H.; Yeka A.; Kamya M. R.; Talisuna A.; Banek K.; Bakyaita N.; Rwakimari J. B.; Rosenthal P. J.; Wabwire-Mangen F.; Dorsey G.; Staedke S. G. Artemisinin combination therapies for treatment of uncomplicated malaria in Uganda. PLoS Clin. Trials 2006, 1 (1), e7 10.1371/journal.pctr.0010007. 16871329 PMC1488893

[ref12] Burckhalter J. H.; Tendick F. H.; Jones E. M.; Jones P. A.; Holcomb W. F.; Rawlins A. L. Aminoalkylphenols as antimalarials. II. (Heterocyclic amino)-α-amino-ο-cresols. The synthesis of camoquin. J. Am. Chem. Soc. 1948, 70, 1363–1373. 10.1021/ja01184a023. 18915746

[ref13] Burckhalter J. H.; Jones E. M.; Rawlins A. L.; Tendick F. H.; Holcomb W. F. U.S. Patent 2,474,819 A, 1949.

[ref14] Burckhalter J. H.; DeWald H. A.; Tendick F. H. An Alternate Synthesis of Camoquin. J. Am. Chem. Soc. 1950, 72, 1024–1025. 10.1021/ja01158a504.

[ref15] Price C. C.; Roberts R. M. The Synthesis of 4-Hydroxyquinolines. 1 I. Through Ethoxymethylenemalonic Ester. J. Am. Chem. Soc. 1946, 68, 1204–1208. 10.1021/ja01211a020. 20990951

[ref16] Price C. C.; Roberts R. M.; Herbrandson H. F. Method for producing 4-hydroxyquinolines. U.S. Patent 2,504,875 A, 1950.

[ref17] Price C. C.; Roberts R. M. Process for making intermediates for producing basic compounds. U.S. Patent 2,614,121 A, 1952.

[ref18] Adaway T. J.; Budd J. T.; King I. R.; Krumel K. L.; Kershner L. D.; Maurer J. L.; Olmstead T. A.; Roth G. A.; Tai J. J.; Hadd M. A. Process for the preparation of halo-4-phenoxyquinolines. WO 1998033774 A1, 1999.

[ref19] Armarego W. L. F. Purification of Laboratory Chemicals, 8th ed.; Elsevier, 2017.

[ref20] Robert J.; Montmorency J. W.; Neuilly-sur S.; Bernard G. Process for the preparation of chlorinated quinolines. U.S. Patent 3,567,732 A, 1971.

[ref21] Conteh L.; Patouillard E.; Kweku M.; Legood R.; Greenwood B.; Chandramohan D. Cost Effectiveness of Seasonal Intermittent Preventive Treatment Using Amodiaquine & Artesunate or Sulphadoxine-Pyrimethamine in Ghanaian Children. PLoS One 2010, 5 (8), e12223 10.1371/journal.pone.0012223. 20808923 PMC2923188

[ref22] Koller G.; Fischer U.; Hungerbühler K. Assessing safety, health, and environmental impact early during process development. Ind. Eng. Chem. Res. 2000, 39 (4), 960–972. 10.1021/ie990669i.

[ref23] Dach R.; Song J. J.; Roschangar F.; Samstag W.; Senanayake C. H. The Eight Criteria Defining a Good Chemical Manufacturing Process. Org. Process Res. Dev. 2012, 16 (11), 1697–1706. 10.1021/op300144g.

[ref24] Gohain M.; Malefo M. S.; Kunyane P.; Scholtz C.; Baruah S.; Zitha A.; Klashorst G. v. d.; Malan H. Process Development for the Manufacture of the Antimalarial Amodiaquine Dihydrochloride Dihydrate. ChemRxiv 2023, 10.26434/chemrxiv-2023-5sw1f. PMC10804403 38268771

[ref25] Cue B. W. Contemporary Chemical Approaches for Green and Sustainable Drugs; Elsevier, 2022; pp 307–331.

[ref26] Szymkuć S.; Gajewska E. P.; Molga K.; Wołos A.; Roszak R.; Beker W.; Moskal M.; Dittwald P.; Grzybowski B. A. Computer-generated “synthetic contingency” plans at times of logistics and supply problems: scenarios for hydroxychloroquine and remdesivir. Chem. Sci. 2020, 11 (26), 6736–6744. 10.1039/D0SC01799J. 33033595 PMC7500088

[ref27] Cutler R. A.; Surrey A. R. The reaction of 4, 7-dichloroquinoline with acetic acid. J. Am. Chem. Soc. 1950, 72 (8), 3394–3395. 10.1021/ja01164a021.

[ref28] Wang J.; Li P.; Shen Q.; Song G. Concise synthesis of aromatic tertiary amines via a double Petasis-borono Mannich reaction of aromatic amines, formaldehyde, and organoboronic acids. Tetrahedron Lett. 2014, 55 (29), 3888–3891. 10.1016/j.tetlet.2014.03.131.

[ref29] Price C. C.; Roberts R. M. 4,7-dichloroquinoline. Org. Synth. 1948, 28, 38 10.15227/orgsyn.028.0038.

