# Supplementary material for: Process Development for the Manufacture of the Antimalarial Amodiaquine Dihydrochloride Dihydrate
Source: Org Process Res Dev. 2023 Dec 18;28(1):124–31. doi: 10.1021/acs.oprd.3c00205 (PMC10804403; doi:10.1021/acs.oprd.3c00205)
Supplement: Supplementary file 1 — op3c00205_si_001.pdf [file op3c00205_si_001.pdf]

Supporting information

Process Development for the Manufacture of the Antimalarial Amodiaquine Dihydrochloride Dihydrate

Mukut Gohain*, Modibo S Malefo, Phaladi Kunyane, Chantal Scholtz, Sangeeta Baruah, Andile Zitha, Gerrit van der Klashorst, Hannes Malan*

* Corresponding authors:

Department of Research and Development at Chemical Process Technologies (Pty) Ltd, 45 Battery Crescent, Waltloo, City of Tshwane, Gauteng, South Africa, 0184.

Email address: mukut@chemprotech.co.za; hannes@chemprotech.co.za

Table of Contents

**Entry**	**Contents**	**Page No.**
Figure S1 – S2	GC chromatograms for compound **5**	3
Figure S3	UV-vis spectrum for compound **3**	4
Figure S4-S5	HPLC chromatograms for compound **3**	4-5
Figure S6	FT-IR spectra for compound **3**	5
Table S1	Thermogravimetric characteristics of compound **3**	6
Figure S7	Thermogravimetric analysis of compound **3**	6
1	Spectroscopic data of compounds **5**. **12**, **14**, **3**	7-8
Figure S8-S10	^{1}H, ^{13}C, COSY NMR data for compound **5**	9-10
Figure S11-S13	^{1}H, ^{13}C, COSY NMR data for compound **12**	10-11
Figure S14-S15	^{1}H, ^{13}C NMR data for compound **14**	12-13
Figure S16-S17	^{1}H, ^{13}C NMR data for compound **3**	14-15
2	Costing and PMI calculations	17-19
3	Abbreviations	20-22

Figure S1: GC chromatogram of crude 4,7-dichloroquinoline (**5**).

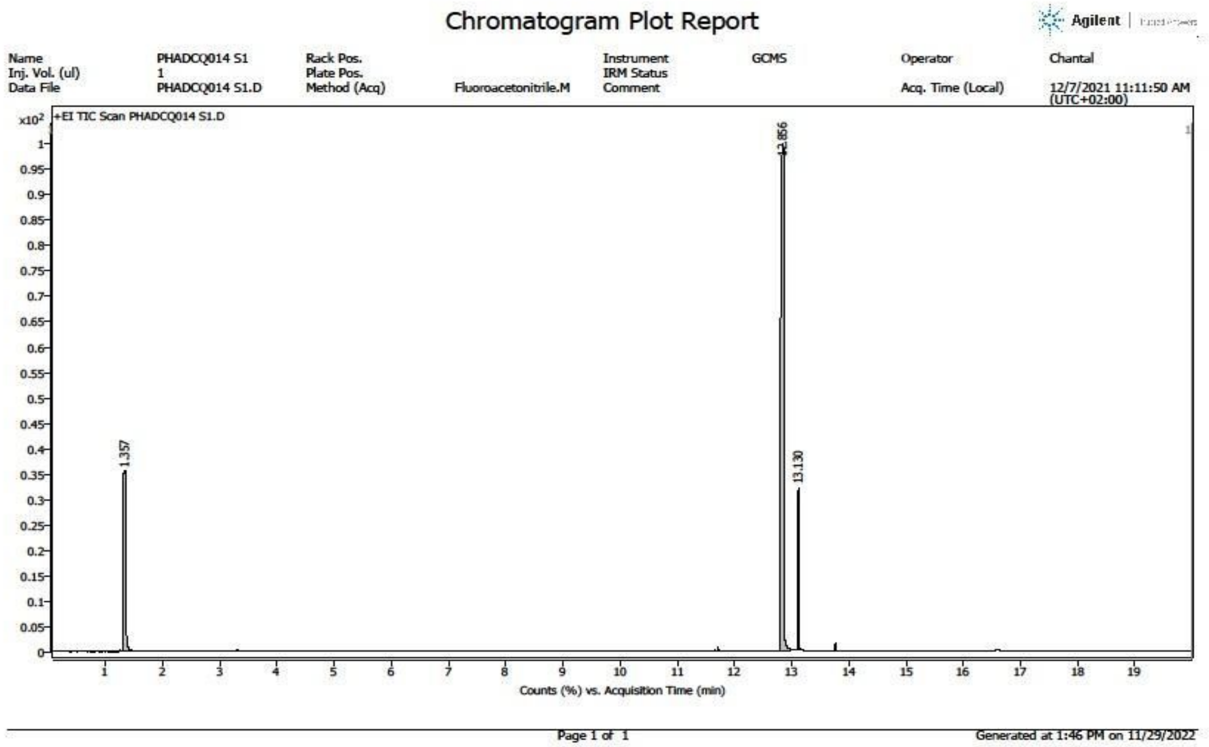

Figure S2: GC chromatogram of 4,7-dichloroquinoline (**5**).

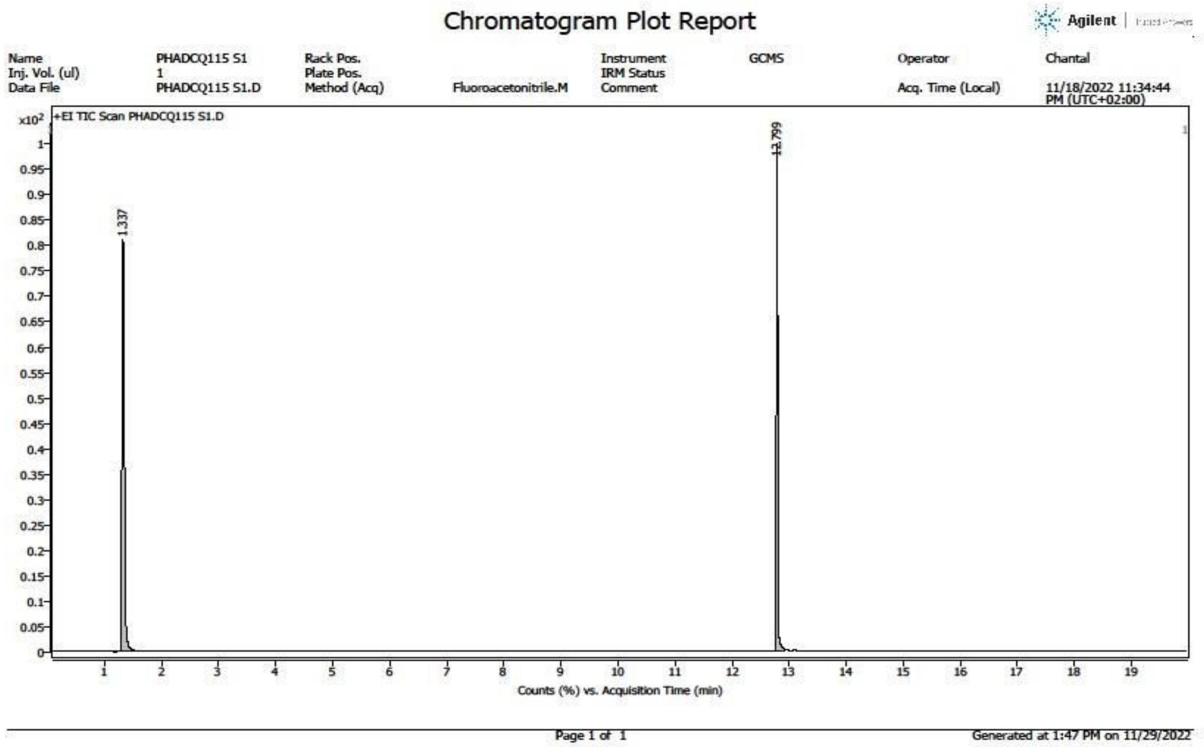

Figure S3: Comparison of UV-Vis Spectrum of USP ADQ (**3**) standard (blue) and CPT synthesised ADQ (**3**, black).

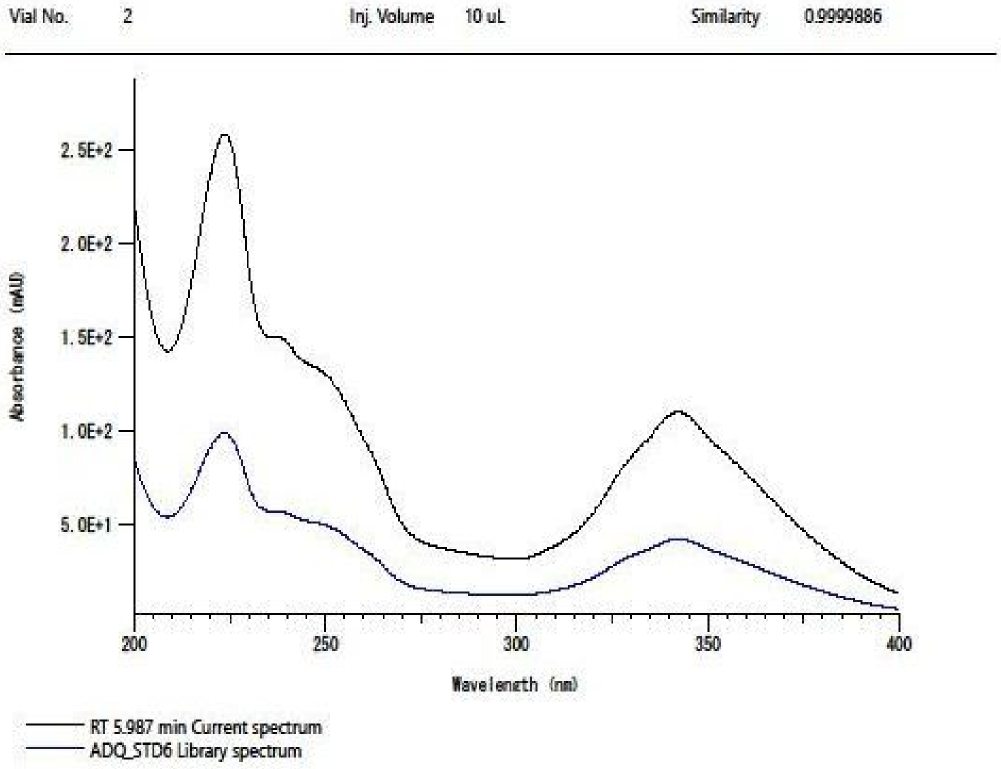

Figure S4: ADQ (**3**) USP standard HPLC chromatogram with report.

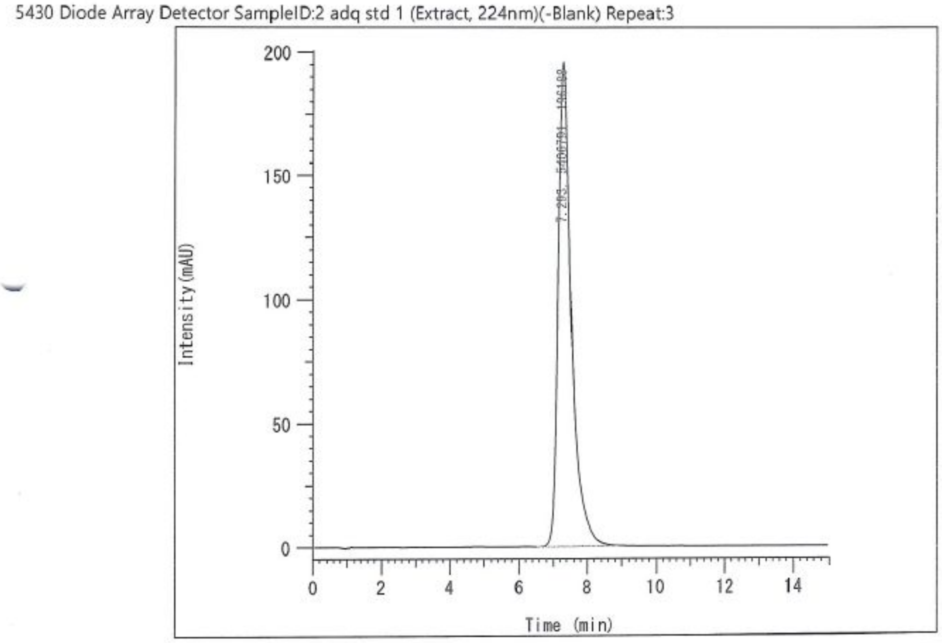

No.	Compounds	RT	Area	Area%	Height	Height%	Concentration	N	Intensity	FWHM	RRT	CRT
1	Peak 1	7.293	5406791	100.000	196188	100.000	---	1862	196	0.40	---	---
			5406791	100.000	196188	100.000						

Figure S5: CPT synthesized ADQ (**3**) HPLC chromatogram with report.

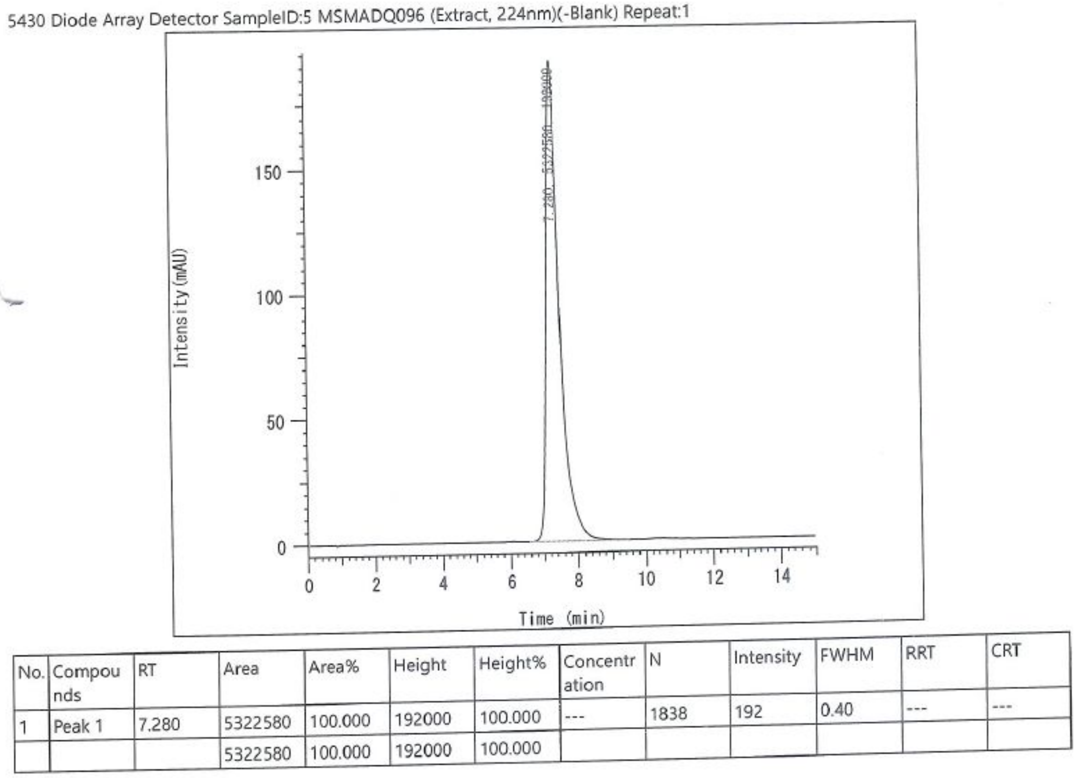

No.	Compounds	RT	Area	Area%	Height	Height%	Concentration	N	Intensity	FWHM	RRT	CRT
1	Peak 1	7.280	5322580	100.000	192000	100.000	---	1838	192	0.40	---	---
			5322580	100.000	192000	100.000						

Figure S6: Comparison of FT-IR Spectra of CPT synthesized ADQ (**3**) and USP ADQ (**3**) standard (bottom).

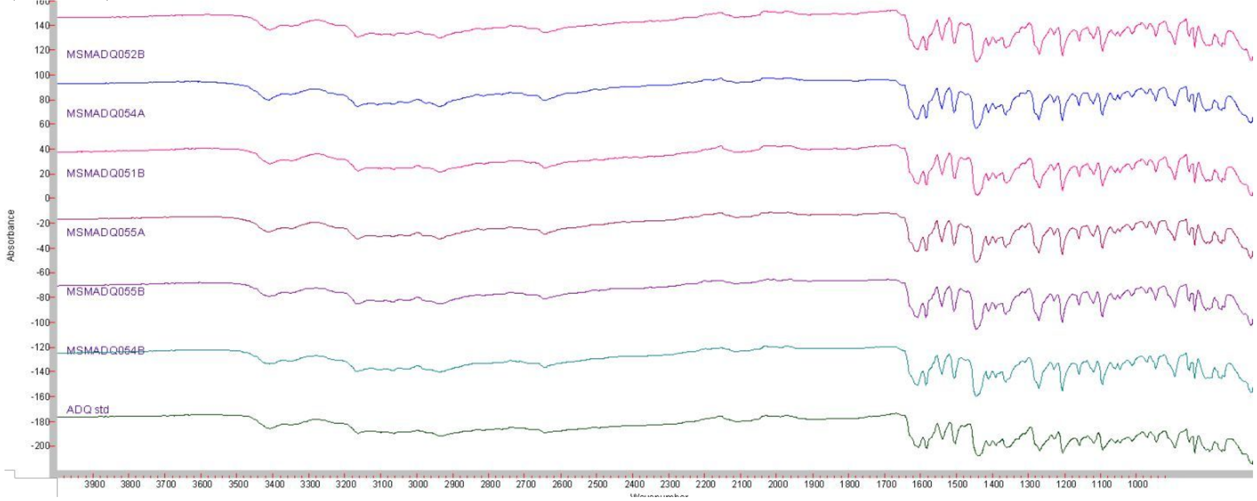

Table S1: Thermogravimetric characteristics of ADQ (**3**) samples.

**Sample ID**	**Temperature range (°C)**	**Mass lost (%)**	**Molecule lost**
ADQ Std	TGA: Experimental<br>T: 41.348 – 150.088	Total:<br>m_i = 99.972<br>m_f = 92.010<br>8.0	$2H_2O$
MSMADQ097C	TGA: Experimental<br>T: 40.111 – 150.079	Total:<br>m_i = 99.869<br>m_f = 92.047<br>8.0	$2H_2O$
MSMADQ095D	TGA: Experimental<br>T: 40.961 – 150.731	Total:<br>m_i = 99.860<br>m_f = 92.067<br>8.0	$2H_2O$

Figure S7: Thermogravimetric analysis of ADQ (**3**) samples.

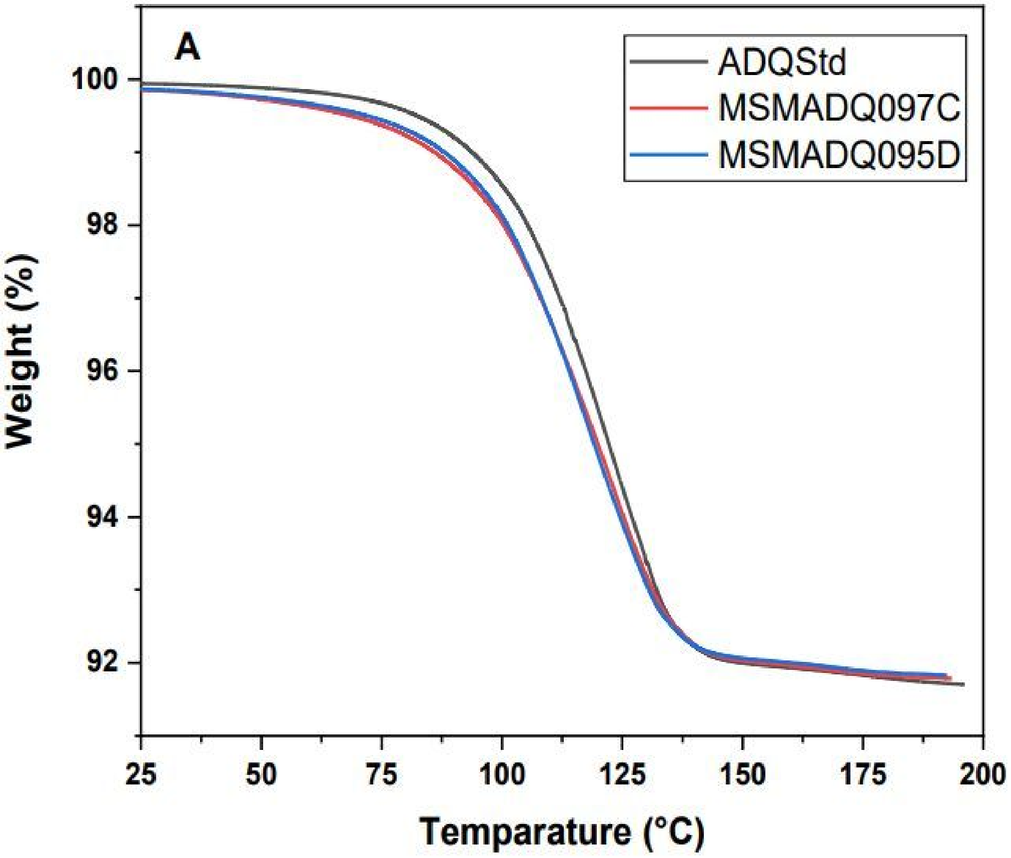

1. **Spectroscopic data of synthesised compounds**

A. **General Information**

All raw materials and solvents purchased were used without further purification. Thin Layer Chromatography (TLC) was performed on Macherey-Nagel 0.2 mm silica gel 60 F254 packed aluminium plates observed under UV light at 254 nm. The synthesised compounds were analysed by FT-IR spectroscopy, NMR spectroscopy on Brucker Avance 500 and 400 MHz instruments with the residual solvent peak as an internal reference (DMSO-d_6 = 2.50 and 39.5 ppm and $CDCl_3$ = 7.26 and 77.16 ppm for ^{1}H and ^{13}C NMR spectra respectively), and Gas Chromatography Mass Spectroscopy (GC-MS). The purity of the final product **3** was determined using High-Performance Liquid Chromatography (HPLC) on a Hitachi system equipped with a LUNA C18 column and a diode array detector set at 224 nm.

Thermal analyses of final ADQ products (**3**) were conducted using thermo-gravimetric analyses (TGA), the TGA-TA 5500 and differential scanning calorimetry (DSC), DSC-TA 2500, under a nitrogen atmosphere. The TGA and DSC thermograms were analysed by TRIOS 5.3.0.48151 version and Origin2018. Isothermal experiments were performed with a TRIOS 5.3.0.48151 version calorimeter with a nitrogen flow rate of 50 mL/min.

4,7-Dichloroquinoline (5)

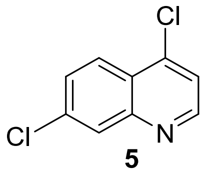

5

^{1}H NMR (400 MHz, $CDCl_3$) δ 8.77 (d, *J* 4.72 Hz, 1H, H-2); 8.15 (d, *J* 8.96 Hz, 1H, H-5); 8.10 (d, *J* 2.04 Hz, 1H, H-8); 7.57 (dd, J_1 8.96, J_2 2.08 Hz, 1H, H-6); 7.47 (d, *J* 4.72 Hz, 1H, H-3). ^{13}C NMR (100 MHz, $CDCl_3$) δ 151.11; 149.55; 142.80; 136.63; 128.87; 128.77; 125.71; 125.12; 121.53.

4,5-Dichloroquinoline (12)

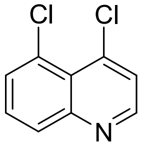

12

^1H NMR (400 MHz, $CDCl_3$) δ 8.72 (d, *J* 4.68 Hz, 1H, H-2)); 8.05 (dd, J_1 8.32 J_2 1.44 Hz, 1H, H-8); 7.67 (dd, J_1 7.56 J_2 1.42 Hz, 1H, H-6); 7.60 (dd, J_1 7.96 J_2 7.96 Hz, 1H, H-7); 7.52 (d, *J* 4.68 Hz, 1H, H-3). ^{13}C NMR (100 MHz, $CDCl_3$) δ 151.30; 149.97; 141.70; 131.09; 130.26; 130.09; 129.61; 125.09; 124.03.

4-Acetamido-2-(diethylaminomethyl)phenol (14)

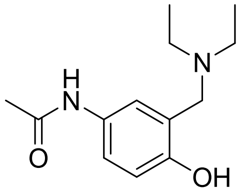

14

^1H NMR (500 MHz, DMSO-d_6) δ 9.63 (s, 1H, NH); 7.28 (s, 1H, Ar-H); 7.25 (d, *J* 8.60 Hz, 1H, Ar-H); 6.60 (d, 1H, *J* 8.60 Hz, Ar-H); 3.65 (s, 2H, C$\underline{H}_2$NEt$_2$); 3.56 (s, 1H, OH); 2.53 (q, 4H, *J* 7.10 Hz, NC$\underline{H}_2$CH$_3$); 1.97 (s, 3H, AcC$\underline{H}_3$); 1.01 (t, 6H, *J* 7.15 Hz, N(CH$_2$C$\underline{H}_3$)$_2$). ^{13}C NMR (125 MHz, DMSO-d_6) δ 167.45; 153.19; 130.82; 122.61; 120.00; 119.34; 115.05; 55.23; 45.80; 23.73; 11.11.

Amodiaquine dihydrochloride dihydrate (3)

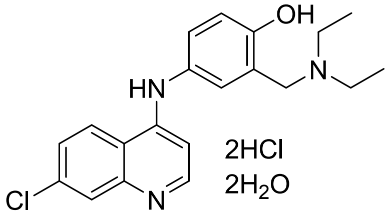

3

^1H NMR (400 MHz, DMSO-d_6) δ 14.88 (br s, 1H, OH); 11.22 (s, 1H, NH); 10.92 (br s, 1H, NH); 10.34 (br s, 1H, NH); 8.94 (d, 1H, *J* 9.20 Hz, Ar-H); 8.47 (d, 1H, *J* 7.08 Hz, Ar-H); 8.19 (d, 1H, *J* 2.08 Hz, Ar-H); 7.83 (dd, 1H, J_1 2.10, J_2 9.10 Hz, Ar-H); 7.69 (d, 1H, *J* 2.56 Hz, Ar-H); 7.37 (dd, 1H, J_1 2.60, J_2 8.68 Hz, Ar-H); 7.22 (d, 1H, *J* 8.68 Hz, Ar-H); 6.84 (d, 1H, *J* 7.04 Hz, Ar-H); 4.24 (s, 2H, CH$_2$); 3.11 (s, 4H, 2x NC$\underline{H}_2$CH$_3$); 1.29 (t, 6H, *J* 7.20 Hz 2x NCH$_2$C$\underline{H}_3$). ^{13}C NMR (100 MHz, DMSO-d_6) δ 156.11; 154.95; 143.09; 138.97; 138.25; 130.13; 128.45; 127.95; 127.20; 126.18; 117.65; 116.87; 115.67; 100.45; 49.14; 46.19; 8.45.

Figure S8: ^{1}H NMR (400 MHz, $CDCl_3$) spectrum of **4,7-Dichloroquinoline** (**5**).

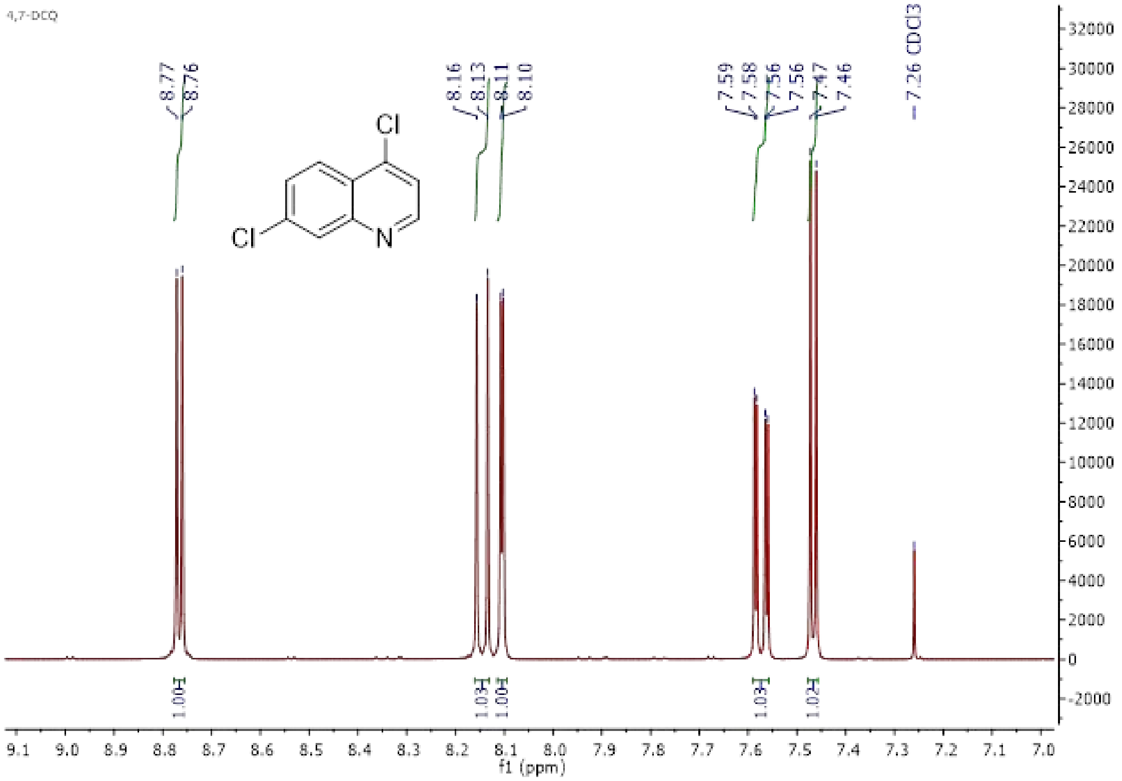

Figure S9: ^{13}C NMR (100 MHz, $CDCl_3$) spectrum of **4,7-Dichloroquinoline** (**5**).

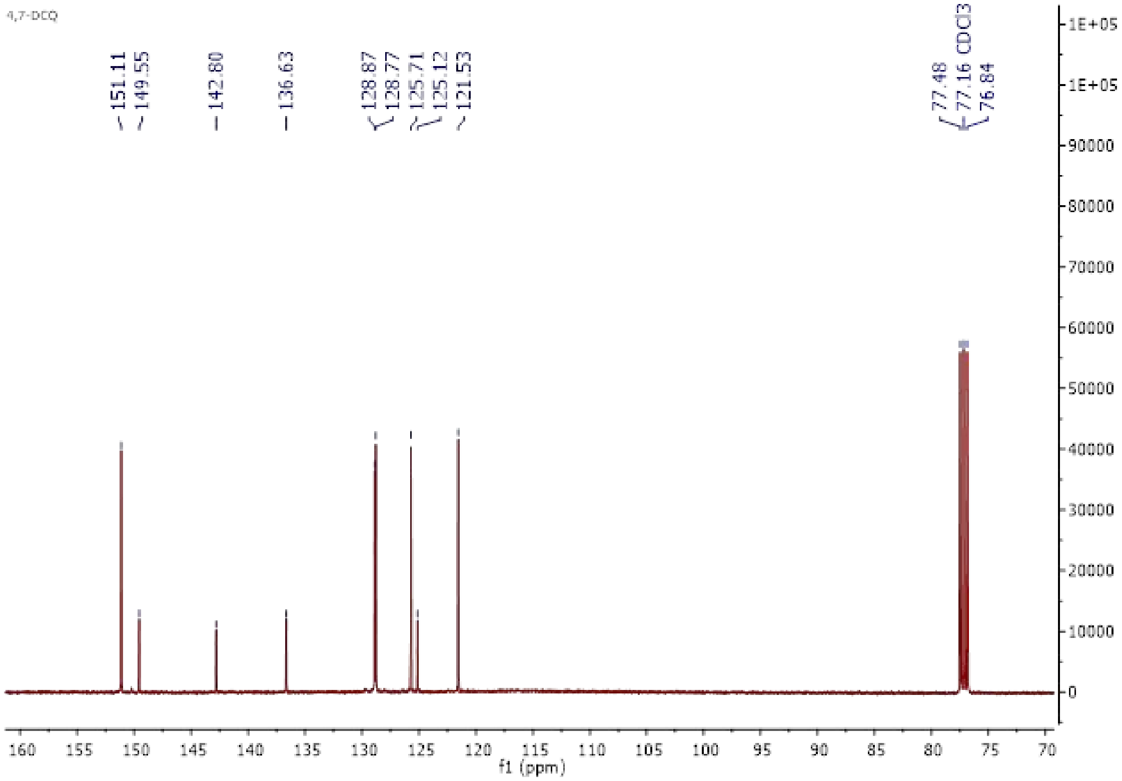

Figure S10: COSY NMR (400 MHz, $CDCl_3$) spectrum of **4,7-Dichloroquinoline** (**5**).

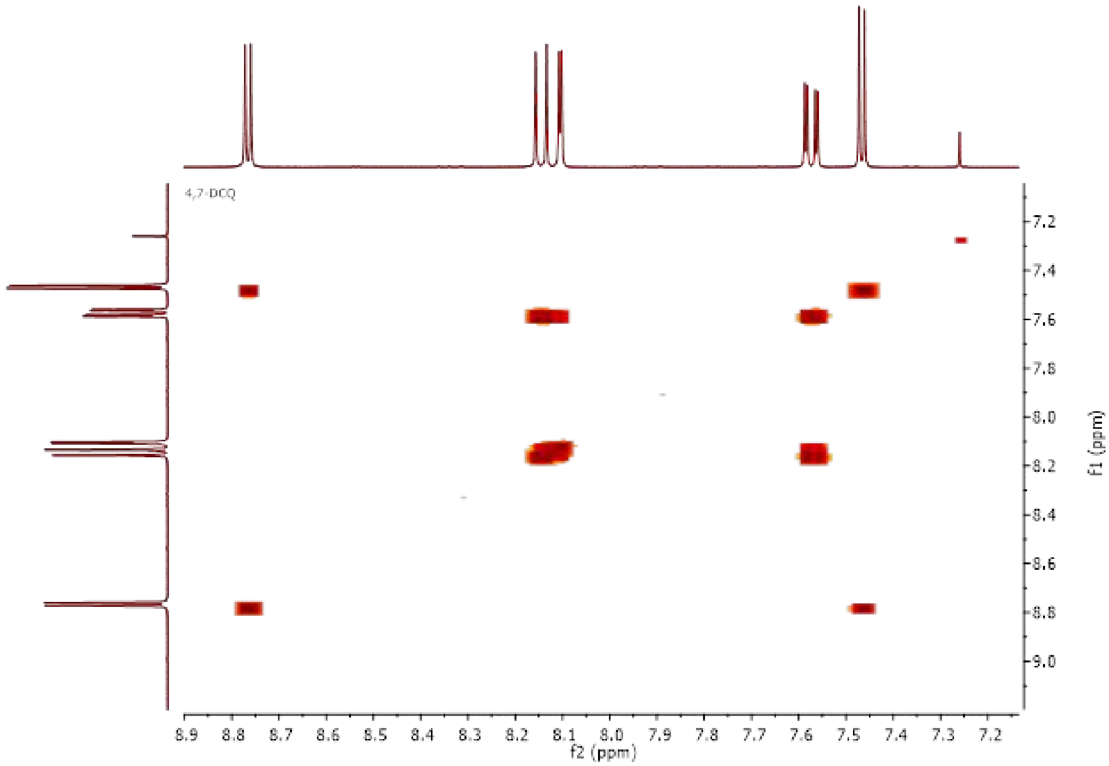

Figure S11: ^{1}H NMR (400 MHz, $CDCl_3$) spectrum of **4,5-Dichloroquinoline** (**12**).

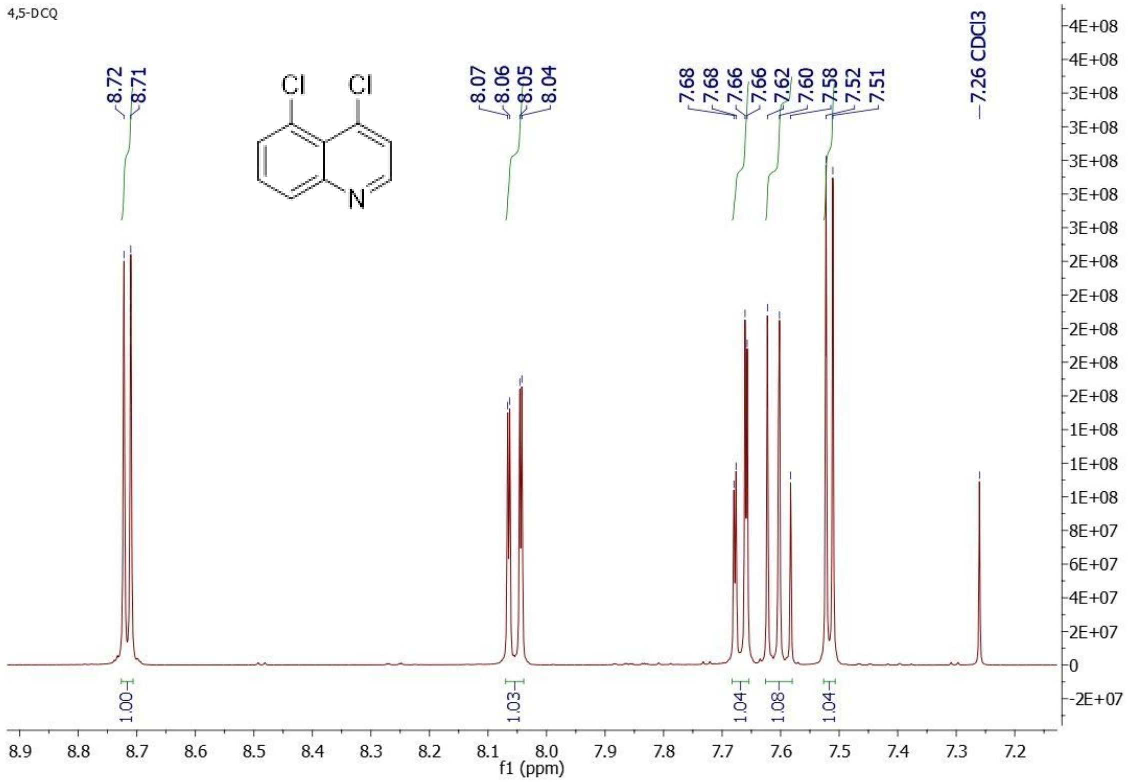

Figure S12: ^{13}C NMR (100 MHz, $CDCl_3$) spectrum of **4,5-Dichloroquinoline** (**12**).

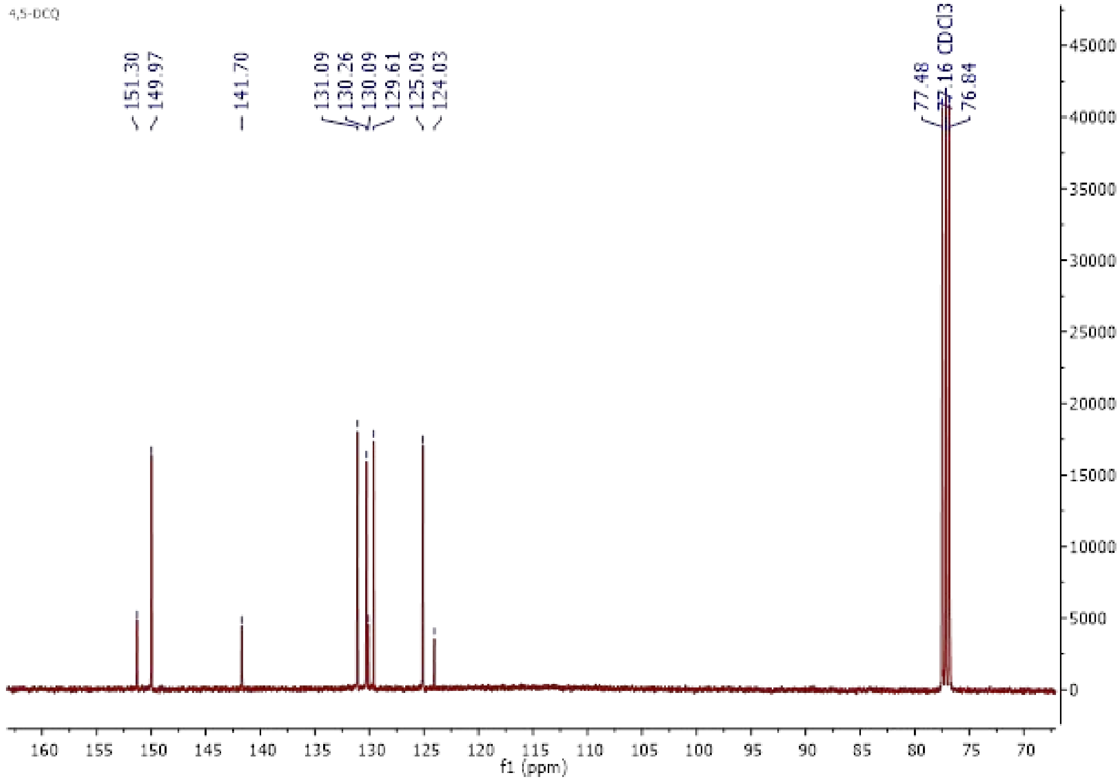

Figure S13: COSY NMR (400 MHz, $CDCl_3$) spectrum of **4,5-Dichloroquinoline** (**12**).

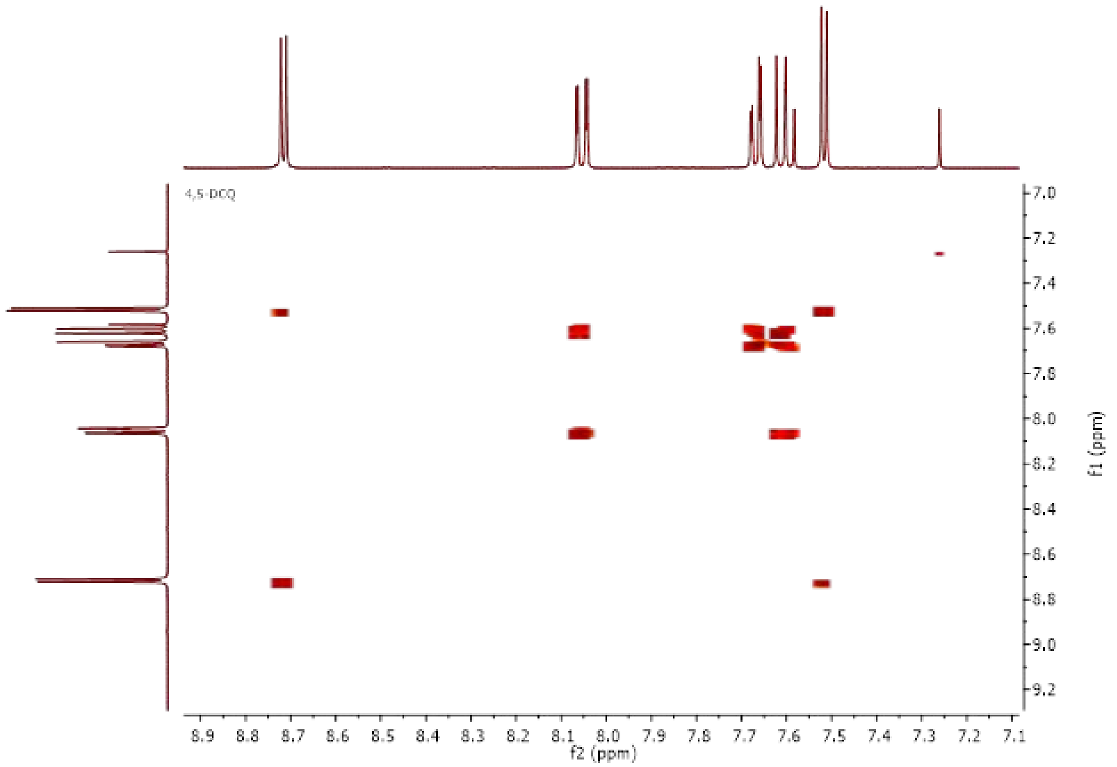

Figure S14: ^{1}H NMR (500 MHz, DMSO-*d6*) spectrum of **4-Acetamido-2-(diethylaminomethyl) phenol** (**14**).

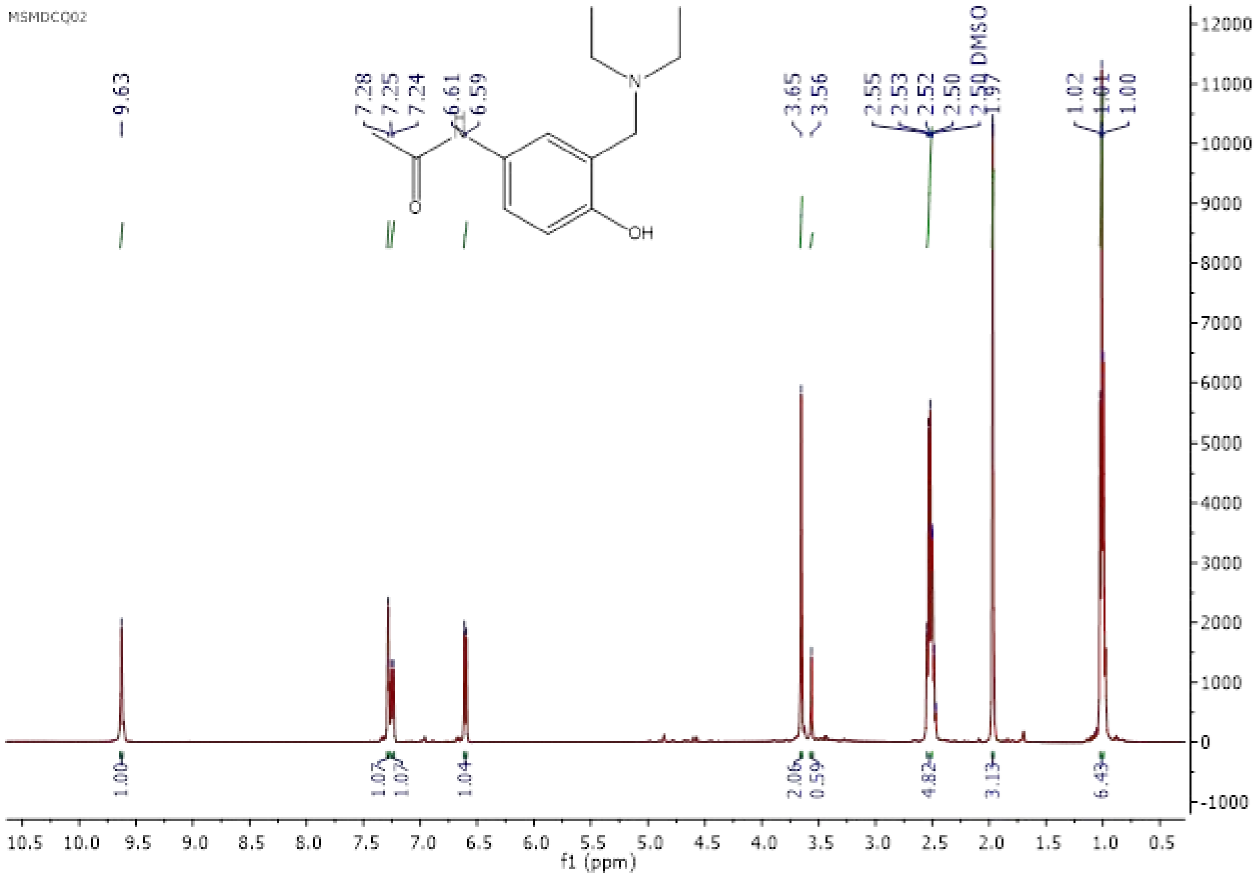

Figure S15: ^{13}C NMR (125 MHz, DMSO-d_6) spectrum of **4-Acetamido-2-(diethylaminomethyl) phenol** (**14**).

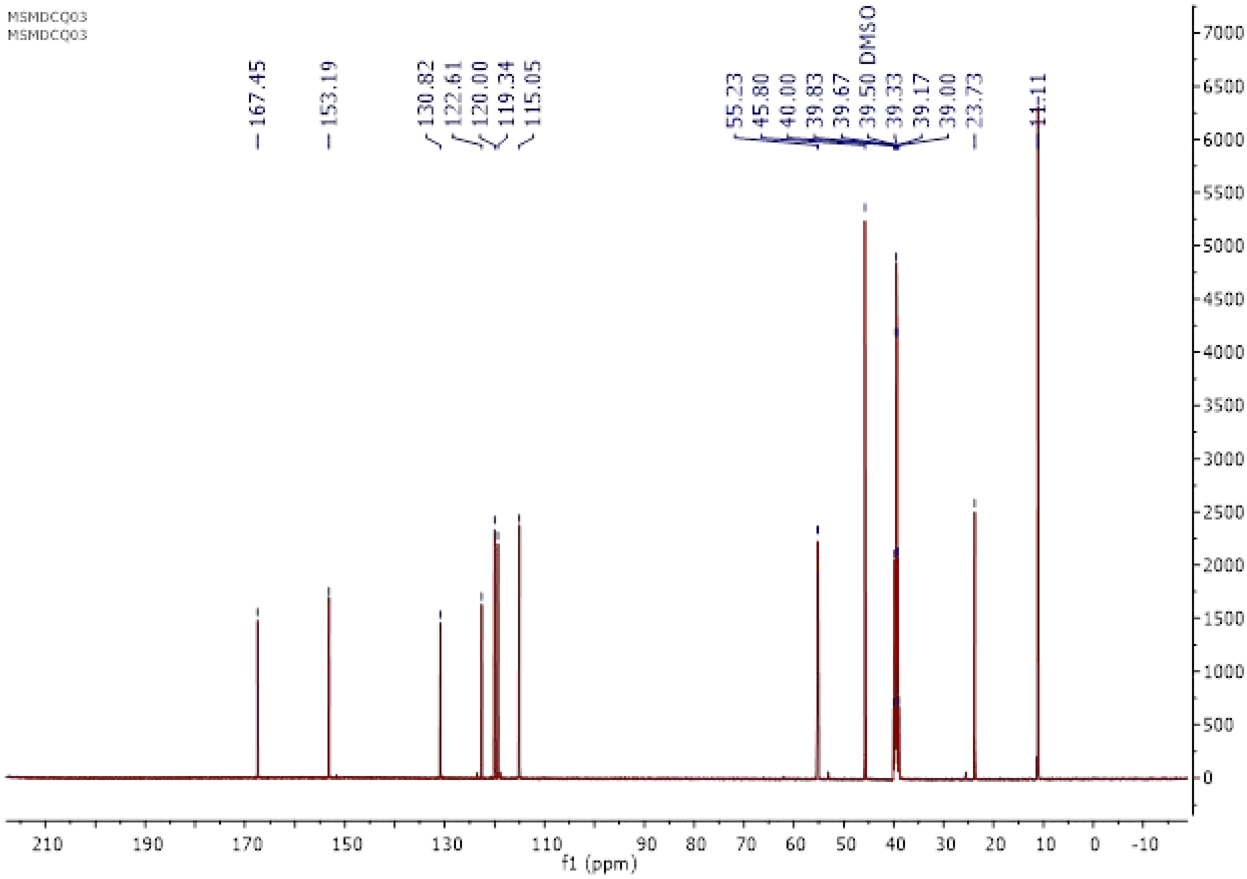

Figure S16: ^{1}H NMR (400 MHz, DMSO-*d*6) spectrum of **Amodiaquine dihydrochloride dihydrate** (**ADQ, 3**).

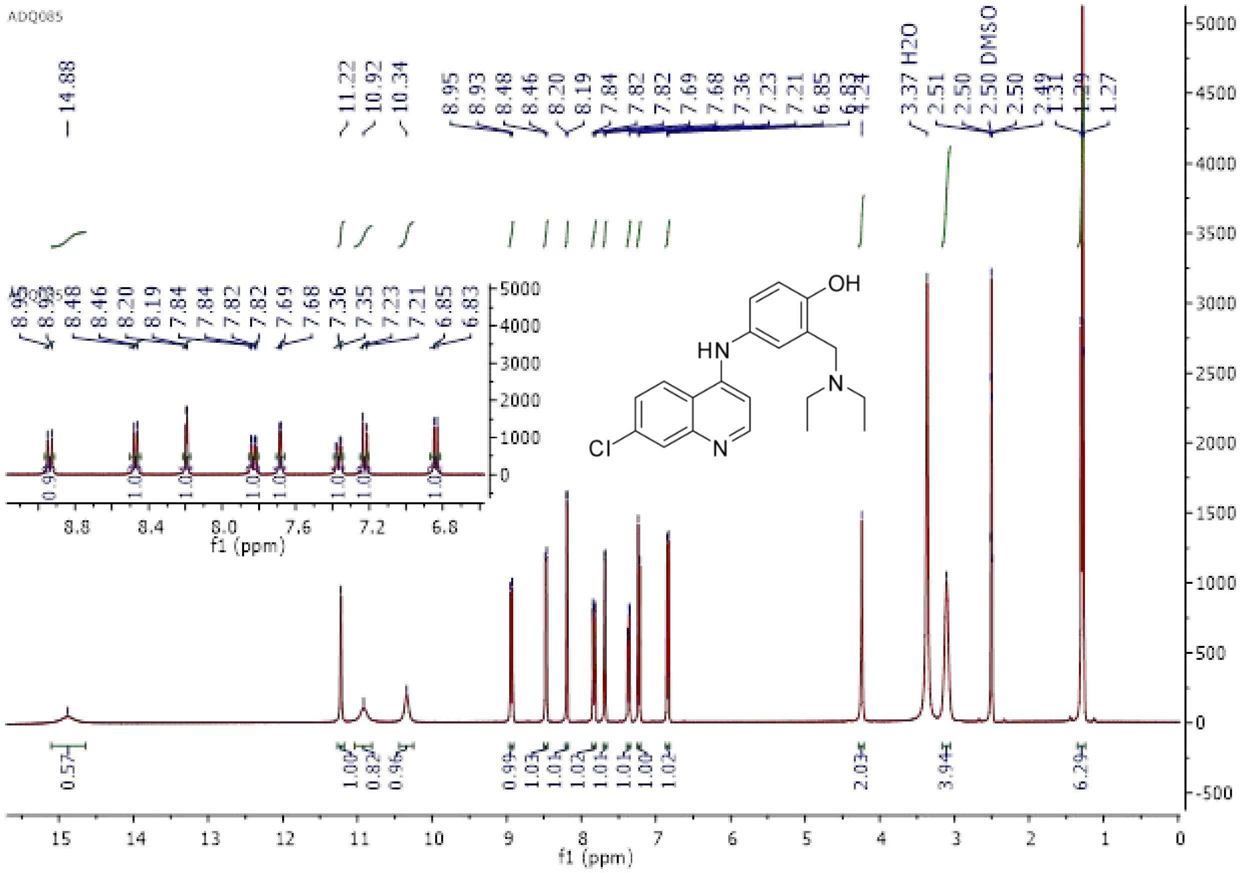

Figure S17: ^{13}C NMR (100 MHz, DMSO-d_6) spectrum of **Amodiaquine dihydrochloride dihydrate** (**ADQ, 3**).

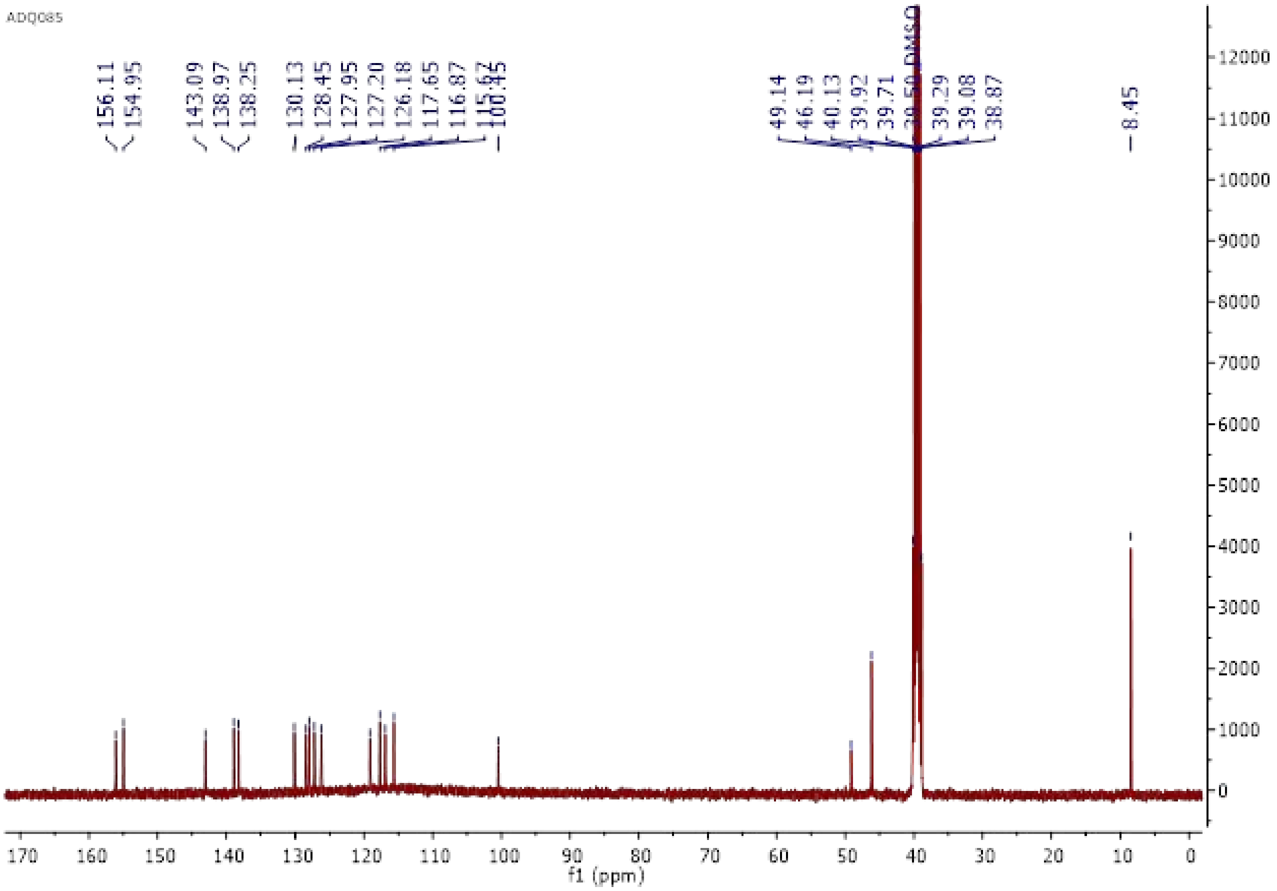

2. Costing and PMI calculations

Reaction	Materials				Mol%	Amount	Amount/		Kg RM/	RM Price	RM Cost $	Cost %
Step		CAS NO	Mol. Wt.	Density	(Volume)	Kg for 100%	Kg Product	Kg RM for 100%	Kg Product	$/kg	/Kg Product	
	3-Chloroaniline	108-42-9	128.00		1.00	128.00	0.86	128.00		4.35	3.73	15.45%
**Step 1 & 2**	Diethylethoxymethylenemalonate	87-13-8	216.00	1.07	1.03	222.48	1.49	222.48		9.00	13.42	55.58%
	Diphenyl ether	101-84-8	170.00	1.08	8.00	55.30	0.37	55.30		3.50	1.30	5.37%
	Toluene	108-88-3	92.00	0.87	6.70	74.35	0.50	74.35		0.99	0.49	2.04%
	Ethyl-7-chloro-4-hydroxyquinoline-3-carboxylate	16600-22-9	252.00									
92%	Product of Step 1		252.00			231.84	1.55	480.13	2.07	0.00		78.45%
**Step 3**	Ethyl-7-chloro-4-hydroxyquinoline-3-carboxylate		252.00		1.00	252.00	1.55	521.88				
	NaOH (25%)	1310-73-2	40.00	1.28	3.70	298.37	1.84	298.37		0.09	0.17	0.69%
	Water for 25% NaOH		18.02	1.00		89.51	0.55	89.51		0.10	0.06	0.23%
	H_2SO_4(10%)	7664-93-9	98.00	1.07	2.50	67.41	0.42	67.41		0.30	0.12	0.52%
	Water for 10% H_2SO_4		18.02			60.67	0.37	60.67		0.10	0.04	0.15%
	7-chloro-4-hydroxyquinoline-3-carboxylic acid	86-47-5	224.00									
91%	Product of Step 2					203.84	1.26	1037.84	5.09			1.59%
**Step 4**	7-chloro-4-hydroxyquinoline-3-carboxylic acid	86-47-5	224.00		1.00	224.00	1.26	1140.48				
	Diphenyl Ether	101-84-8	170.00	1.08	5.00	60.48	0.34	60.48		3.50	1.19	4.92%

	$POCl_3$	10025-87-3	153.00	1.65	1.00	153.00	0.86	153.00		3.25	2.79	11.55%
	HCl (10%)	7647-01-0	36.50	1.05	1.30	30.58	0.17	30.58		0.30	0.05	0.21%
	Water for 10% HCl		18.02			27.52	0.15	27.52		0.10	0.02	0.06%
	NaOH (25%)	1310-73-2	40.00	1.28	1.20	86.02	0.48	86.02		0.95	0.46	1.90%
	Water for 25% NaOH		18.02			25.80	0.14	25.80		0.10	0.01	0.06%
	Toluene	108-88-3	92.00	0.87	2.00	38.84	0.22	38.84		0.99	0.22	0.89%
	H_2O		18.02	1.00	7.00	156.80	0.88	156.80		0.10	0.09	0.36%
	Product of Step 3: 4,7-Dichloroquinoline	86-98-6	198.00									
90%	Product of Step 4: 4,7-DCQ					178.20	1.00	1719.52	9.65		24.15	19.97%
									9.65			100.00%

Overall yield (%)	75%
Diphenyl Ether Recovery	95%
Solvent and water Recovery	90%

	$/kg	Raw Material Margin
Raw Material cost	24.15	
Target Price: 4,7-DCQ	42.00	43%

PMI
ALL RMs With recycling of solvents and water 9.65

Reaction	Materials				Mol%	Amount	Amount	Amount	Kg RM/	RM Price	RM Cost $	Cost %
step	Mannich Base	CAS NO	Mol. Wt.	Density	(Volume)	Kg RM for 100%	/ Kg Product	Kg RM	Kg Product	$/Kg	/Kg Product	
Step 1	4-Acetamidophenol	103-90-2	151.00		1.00	151.00	0.38	151.00		8.95	3.40	20.52 %
	N,N-Diethylamine	109-89-7	73.10	0.71	1.25	91.38	0.23	91.38		3.00	0.69	4.16 %
	Paraformaldehyde	30525-89-4	30.00		1.20	36.00	0.09	36.00		0.58	0.05	0.32 %
	Toluene	108-88-3	92.00	0.87	4.00	52.37	0.13	52.37		0.99	0.13	0.79 %
	4-Acetamido-2-(diethylaminomethyl)phenol	121-78-8	236.00									
95%	Product of Step 1					224.20	0.56	330.74	1.48			
**Step 2**	4-Acetamido-2-(diethylaminomethyl)phenol	121-78-8	236.00		1.00	236.00	0.56	348.15				
	HCl (32%)		36.50	1.16	2.20	192.73	0.46	192.73		0.30	0.14	0.83 %
	Water for 32% HCl		18.00			40.95	0.10	40.95		0.10	0.01	0.06 %
	4,7-Dichloroquinoline	86-98-6	198.00		1.00	198.00	0.47	198.00		24.15	11.43	68.98 %
	NaOH (25%)		40.00	1.28	2.70	203.90	0.49	203.90		0.95	0.46	2.79 %
	Water for 25% NaOH		18.00			61.17	0.15	61.17		0.10	0.01	0.24 %
	Water		18.00	1.00	7.14	168.50	0.40	168.50		0.10	0.04	0.24 %
	Amodiaquine dihydrochloride dihydrate	6398-98-7	464.80									
100%	Product of Step 4					464.80	1.11	1213.41	2.61			

**Step 3**	Amodiaquine dihydrochloride dihydrate	6398-98-7	464.80		1.00	464.80	1.11	1213.41				
Recryst.	Ethanol (83%)		46.00	0.79	1.20	44.01	0.11	44.01		1.40	0.15	0.89%
	HCl (32%)		36.50	1.08	0.25	40.16	0.10	40.16		0.30	0.03	0.17%
	Water for 32% HCl		18.00			8.53	0.02	8.53		0.10	0.00	0.01%
	Water		18.00	1.00	2.00	92.96	0.22	92.96		0.10	0.02	0.13%
	Amodiaquine dihydrochloride dihydrate	6398-98-7	464.80									
90%	Product of step 4: ADQ·2HCl·2H_2O:		464.80			418.32	1.00	1399.07	3.34		16.57	100%
									3.34			
									PMI			

Overall Yield (%)	86%
Diphenyl ether recovery	95.00%
Solvent and water recycle	90.00%

	$/kg	RM Margin:	ALL RMs
Raw material cost:	16.57		With recycling of solvents and water 3.34
CPT sourced price of ADQ USP Grade	40.00	59%	

3. Abbreviations

AcOH	-	acetic acid
ACT	-	artemisinin-based combination therapy
ADQ	-	amodiaquine dihydrochloride dihydrate
APIs	-	active pharmaceutical ingredients
Aq.	-	aqueous
$CDCl_3$	-	deuterated chloroform
CPT	-	Chemical Process Technologies (Pty) Ltd
CQ	-	chloroquinoline acid
4,7-DCQ	-	4,7-dichloroquinoline
DEA	-	diethylamine
DMSO	-	dimethyl sulfoxide
DPE	-	diphenyl ether
DSC	-	differential scanning calorimetry
EtOAc	-	ethyl acetate
EtOH	-	ethanol
Equiv.	-	equivalent
FT-IR	-	Fourier transform infrared
GC	-	gas chromatography
GC-MS	-	gas chromatography-mass spectrometry
HCl	-	hydrochloric acid
H_2O	-	water
HPLC	-	high performance liquid chromatography
HRMS	-	high resolution mass spectrometry
H_2SO_4	-	sulphuric acid
IPA	-	isopropanol
IR	-	infrared
MeOH	-	methanol
mp	-	melting point
NMR	-	nuclear magnetic resonance
NaOH	-	sodium hydroxide
PMI	-	process mass intensity

$POCl_3$	-	phosphoryl chloride
TGA	-	thermogravimetric analysis
TLC	-	thin layer chromatography
p-TSA	-	*para*-toluenesulfonic acid
USP	-	United States Pharmacopeia
UV	-	ultraviolet
WHO	-	World Health Organization
